# Inflammatory macrophage to hepatocyte signals can be prevented by extracellular vesicle reprogramming

**DOI:** 10.1242/jcs.260691

**Published:** 2023-05-12

**Authors:** Priyanka Ghosh, Kyo Sasaki, Isabel Aranzazu Pulido Ruiz, Kayla E. King, Steven A. Weinman, Ann L. Wozniak

**Affiliations:** ^1^ Department of Internal Medicine, University of Kansas Medical Center, Kansas City, KS 66160, USA; ^2^ Liver Center, University of Kansas Medical Center, Kansas City, KS 66160, USA

**Keywords:** Trafficking, Cellular crosstalk, Extracellular vesicles, RILP, Hepatocyte, Macrophage

## Abstract

Macrophage-derived extracellular vesicles (EVs) play key roles in intercellular communication. Within the liver, they have been linked to several inflammatory diseases including nonalcoholic fatty liver disease (NAFLD). In this study, we found that inflammatory macrophages cause injury to hepatocytes, in part by a cell–cell crosstalk phenomenon involving the secretion of EVs containing pro-inflammatory cargo. Incorporation of these inflammatory signals into EV requires the cleavage of the trafficking adaptor protein RILP, which, as previously shown, results from inflammasome-mediated caspase-1 activation. RILP cleavage can be blocked by overexpressing a dominant negative, non-cleavable form of RILP (ncRILP). EV preparations from ncRILP-expressing cells are, by themselves, sufficient to suppress inflammatory effects in hepatocytes. These results suggest that both direct RILP manipulation and/or supplying ncRILP-modified EVs could be used as a novel therapy for the treatment of inflammatory liver diseases.

## Teaser

**Summary:** Manipulation of the Rab7 adaptor protein RILP in macrophages alters extracellular vesicle-mediated cell–cell crosstalk and protects hepatocytes against an injury signal.

## INTRODUCTION

Non-alcoholic fatty liver disease (NAFLD) is emerging as a major cause of morbidity and mortality in the USA and is a leading indication for liver transplantation (Estes et al., 2018). The disease results from hepatocellular fat accumulation which initiates a process of inflammation and fibrosis. However, initial fat deposition alone is not sufficient to drive the disease, and, in patients with a progressive disease, liver macrophages play a key role in driving inflammation, fibrosis and progression to cirrhosis. Although chemokine antagonists that block macrophage recruitment can reduce fibrosis (Krenkel and Tacke, 2017), there are no currently approved pharmacological therapies for inflammatory fatty liver disease. It is therefore important to identify the mechanisms and/or pathways that drive disease progression.

Liver macrophages are crucial for the maintenance of hepatic homeostasis; these cells are highly dynamic in their response to injury. In addition to their well-known function of engulfing microorganisms, macrophages coordinate the injury response between numerous cell types. Many liver pathologies result from chronic disease processes involving inflammation, and, during inflammatory disease states, macrophages become activated and in turn modulate the fate of neighboring cells including hepatocytes (Kazankov et al., 2019). This directly contributes to inflammation and liver injury and, in the case of progressive NAFLD, results in hepatocyte dysfunction ultimately leading to the development of liver fibrosis, cirrhosis and hepatocellular carcinoma.

Inflammasome activation plays a key role in the inflammatory disease process. Inflammasomes are intracellular multiprotein complexes that sense danger signals from damaged cells and pathogens (Davis et al., 2011). The NLRP3 inflammasome (also known as cryopyrin and NALP3) is expressed by myeloid cells and is upregulated in response to macrophage stimulation by pathogen-associated molecule patterns (PAMPs). NLRP3 forms a complex with the adaptor protein apoptosis-associated speck like protein (ASC; also known as PYCARD) to recruit caspase- 1. Once activated, the caspase-1 component of the active inflammasome carries out any number of processes including processing and/or cleavage of cellular proteins such as IL-1β and IL-18. These proteins and cargoes can then be secreted from the cell through several cellular trafficking pathways and further promote inflammation (Gross et al., 2011; Strowig et al., 2012; Szabo and Csak, 2012).

Vesicular transport is controlled by microtubule-dependent kinesin and dynein motor proteins. Within the endo-lysosomal trafficking pathway, vesicle motility occurs in a bidirectional, stop-and-go manner with alternating activities of kinesin-based motors which regulate plus-end movement towards the plasma membrane and dynein-based motors regulating minus-end movement toward the microtubule-organizing center. Rab-interacting lysosomal protein (RILP) is a key regulator of endo-lysosomal trafficking. It interacts with Rab7 (Rab7a and Rab7b forms in mammals) through its C-terminal domain whereas its N-terminal domain recruits the dynein–dynactin motor complex. RILP is thus responsible for directing minus-end directed microtubule transport of Rab7-containing vesicles from the endosome to the multivesicular body (MVB) and finally the lysosome (Cantalupo et al., 2001). Our lab previously identified RILP as a novel target of caspase-1 (Adams et al., 2018). The cleavage of RILP removes the dynein-binding domain and generates a C-terminal fragment of the protein. Cleaved RILP (cRILP) reroutes endocytic vesicular trafficking away from the lysosome and instead the vesicle moves towards the plasma membrane. This leads to an enhancement of plasma membrane fusion events and accounts for a burst of vesicular secretion, including the secretion of EVs (Jones-Jamtgaard et al., 2019; Wozniak et al., 2020, 2016).

Several reports suggest cell-to-cell communication occurs via the transfer of extracellular vesicular cargoes (Alexander et al., 2015; Li et al., 2013). EVs contain a variety of biomolecules including protein and RNA. Cellular stress and inflammation cause dramatic increases in EV secretion and induce changes in the proportions of these cargo molecules (Bala et al., 2011; McDaniel et al., 2014; Thomou et al., 2017). We found that the cleaved form of RILP (cRILP) could influence miRNA cargo loading into the EVs, enriching for miRNAs that are involved in inflammatory processes. Through the manipulation of RILP cleavage, we could show a differential regulation in which miRNAs are specifically secreted within the EVs. Blocking RILP cleavage specifically enhanced for anti-inflammatory miRNAs within the EVs while simultaneously suppressing exosomal miRNAs that are involved in pro-inflammatory processes (Wozniak et al., 2020).

We previously linked inflammasome activation to both increased RILP cleavage and increased EV secretion in several liver inflammatory diseases, including progressive NALFD (Wozniak et al., 2020). Because RILP cleavage influences both the abundance of EV secretion as well as cargo specificity, we hypothesized that RILP cleavage significantly contributes to the promotion and progression of inflammatory liver disease. The aim of this study was to determine the role of RILP cleavage in cell–cell communication during inflammatory disease states. We found that inflammasome activation and subsequent RILP cleavage promotes the secretion of pro-inflammatory EVs that directly influence hepatocyte health.

## RESULTS

### The inflammasome mediates cell–cell crosstalk

**Fig. 1.**
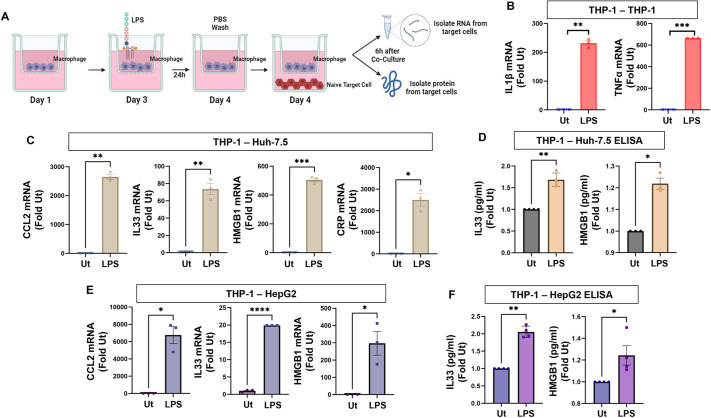
**The inflammasome mediates cell–cell crosstalk.** (A) Schematic of the co-culture system used in these studies. Producer monocytes were plated in the insert of transwell plates and differentiated into macrophage-like cells with PMA. After 3 days, LPS was added at a concentration of 100 ng/ml. After 24 h the cells were washed with PBS to remove any trace of LPS, the insert was transferred to a new well containing naive target cells. After a 6-h incubation, RNA and cell lysates were collected from the target cell. All experiments were performed at least *n*=3 times. (B) Co-culture showing an increase in inflammatory markers in naïve THP1 cells when they were co-cultured with LPS treated THP-1 macrophages. *n*=3. (C) Co-culture of LPS-treated THP-1 cells with target Huh-7.5 hepatocytes showing increased RNA of cell injury markers within the hepatocyte. *n*=3. (D) After co-culturing, Huh-7.5 target hepatocytes were collected, and the intracellular protein content was assessed my ELISA. *n*=3. (E,F) Co-culture of LPS-treated THP-1 cells with target HepG2 hepatocytes showing increased RNA, *n*=3 (E) and protein expression, *n*=4 (F) of cell injury markers within the hepatocyte. Data are shown as mean±s.e.m. *P≤0.05; **P≤0.01; ***P≤0.001; ****P≤0.0001 (two-tailed unpaired Student's *t*-test). Ut, untreated.

To determine the role of the inflammasome in cell–cell crosstalk, we used a co-culture system. Monocytes were plated in the insert of transwell plates and treated with phorbol 12-myristate 13-acetate (PMA) to induce differentiation into macrophage-like cells. We then treated these producer macrophages with lipopolysaccharide (LPS; 100 ng/ml) for 24 h (Dalby, 2018; Monguió-Tortajada et al., 2018). After 24 h, the cells were washed with PBS to remove any traces of LPS, and the inserts were transferred to a new well containing naive target cells. After a 6-h incubation, RNA and cell lysates were collected from the target cells (Fig. 1A). We confirmed inflammasome activation in the producer cells by measuring the amount of mature IL-1β, a direct downstream marker of inflammasome activation. Treatment of THP-1 cells with LPS resulted in a significant increase in both the intracellular and secreted levels of mature IL-1β, thus confirming inflammasome activation in this model (Fig. S1A,B).

We first examined macrophage–macrophage crosstalk. Naïve THP-1 macrophages were co-cultured with producer THP-1 macrophages that had been pre-treated with LPS. We saw a dramatic increase in inflammatory cytokine mRNA for IL-1β and TNFα (also known as TNF) in the naïve cells, demonstrating macrophage to macrophage crosstalk (Fig. 1B). We then asked whether similar crosstalk occurs between macrophages and hepatocytes. Co-culture of LPS-treated THP-1 cells with target hepatocytes (Huh-7.5 or HepG2) increased the expression of several hepatocyte-specific injury markers, such as CCL2, HMGB1, IL-33 and CRP within the hepatocyte (Fig. 1C,E). To confirm that the increases in mRNA correlate to protein expression, we collected the target cell lysates and measured cytokine levels by ELISA (Fig. 1D,F). Taken together, these results define a model by which inflammasome-mediated cell–cell crosstalk can be assessed but also suggest that, when stimulated, macrophages can influence hepatocyte function and lead to the expression of genes that have been associated with injury.

### Blocking RILP cleavage in producer cells imparts a protective effect in target cells

We previously reported that inflammasome activation results in the cleavage of the trafficking adaptor protein RILP (Adams et al., 2018). This cleavage reroutes intracellular trafficking resulting in a pro-secretory phenotype. Therefore, we next determined whether RILP cleavage is required for the generation of an inflammatory cell–cell crosstalk signal to naïve cells. To confirm LPS-mediated RILP cleavage, we performed immunocytochemistry on THP-1 producer cells. Owing to the limited availably of RILP antibodies suitable for immunofluorescence, we elected to use Rab7 as a surrogate for RILP localization and cleavage. Rab7 is a RILP-binding protein that colocalizes with, and follows, RILP (cleaved and non-cleaved) throughout the cell (Adams et al., 2018; Wozniak et al., 2020). Full-length RILP localizes in a tight vesicular structure near the perinuclear region, whereas the cleaved form of RILP re-distributes throughout the cytoplasm. As a proof-of-principle for the use of Rab7 as a surrogate for RILP localization, microglia cells were treated with LPS to stimulate the inflammasome and thus RILP cleavage. They were fixed with 4% PFA, permeabilized with acetone and immunostained for Rab7. In untreated cells, Rab7 localized at the perinuclear region, near the mitotic center (Fig. S2A). This localization was similar to that seen when a non-cleavable RILP, ncRILP, was overexpressed (Fig. S2B, left panel). After inflammasome activation, Rab7 redistributed throughout the cellular periphery and extended to the plasma membrane. This localization is similar to that seen when cRILP is overexpressed (Fig. S2B, right panel). Analysis of these images using ImageJ shows a significant difference in Rab7 cellular distribution between untreated and LPS-treated cells (Fig. S2).

**Fig. 2.**
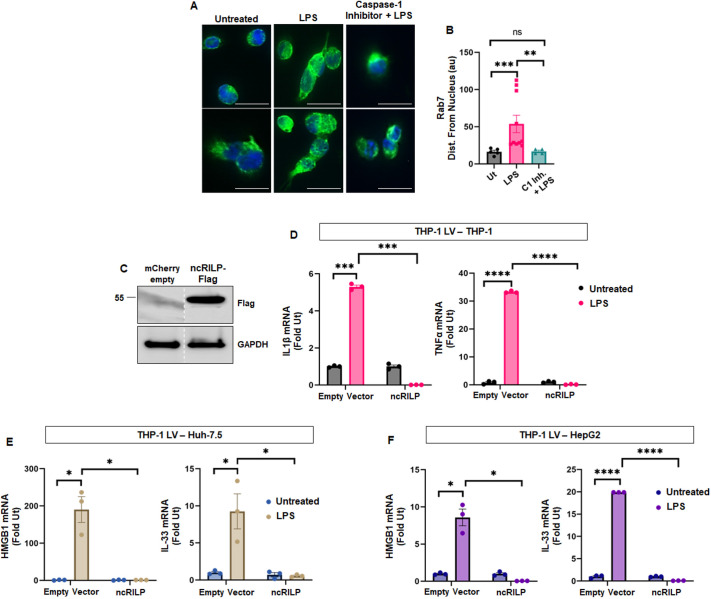
**Blocking RILP cleavage in producer cells imparts a protective effect in target cells.** (A) The localization of the RILP-binding protein Rab7 is a surrogate for RILP cleavage. Producer THP-1 cells were treated with 100 ng/ml LPS for 24 h, fixed with 4% PFA and immunostained for Rab7. LPS treatment results in a redistribution of Rab7 throughout the cell periphery, indicating RILP cleavage. This cleavage is confirmed by incubating LPS-treated cells with the caspase-1 inhibitor Z-YVAD-FMK (100 µM). Scale bars: 15 µm. Ut, untreated. (B) ImageJ analysis quantifying the cellular distribution of Rab7 from the nucleus. *n=*a minimum of 30 cells from three individual experiments*.* (C) Western blot analysis of THP-1 producer cells that were transduced with lentivirus expressing control mCherry (empty vector) or virus expressing ncRILP–Flag. The dotted line represents a cut in the blot. Image representative of three repeats. (D) When co-cultured with LPS treated macrophages, IL-1β and TNFα increase dramatically in target THP-1 cells. Expression of ncRILP in treated macrophage completely blocks this response. *n*=3. (E,F) Similar results occurred in hepatocytes. Hepatocyte-derived pro-inflammatory markers HMGB1 and IL-33 increase in Huh-7.5 (E) and HepG2 (F) when they are co-cultured with inflammatory producer macrophages. These increases were completely blocked when the target cells were co-cultured with ncRILP-expressing producer inflammatory macrophages. *n*=3. All data are shown as mean±s.e.m. *P≤0.05; **P≤0.01; ***P≤0.001; ****P≤0.0001; ns, not significant (two-tailed unpaired Student's *t*-test).

To determine if RILP is cleaved in our THP-1 producer cells, we treated THP-1 cells with LPS and cultured them as stated previously. Immunostaining for Rab7 reveals clear distribution differences of Rab7 (Fig. 2A). In control, untreated conditions, Rab7 is localized to the perinuclear region whereas in THP-1 cells treated with LPS, the Rab7 distribution showed structures extending from this region. ImageJ analysis of Rab7 cellular distribution showed a significant difference between untreated and LPS-treated cells (Fig. 2B). RILP is a direct target of caspase-1 (Adams et al., 2018). The addition of caspase-1 inhibitor Z-YVAD-FMK to LPS-treated cells restored Rab7 distribution to the perinuclear region, further confirming caspase-1-mediated RILP cleavage in producer macrophages.

To determine whether RILP cleavage is required for the transfer of an inflammatory crosstalk signal to naïve cells, we utilized a dominant-negative non-cleavable form of RILP (ncRILP). ncRILP simultaneously binds both Rab7 and dynein, thus preventing the trafficking changes induced by cRILP (Adams et al., 2018; Wozniak et al., 2020). Producer THP-1 monocytes were transduced with mCherry-based lentivirus for empty vector (control) or ncRILP–Flag expression (Fig. 2C). The producer cells were treated with PMA to induce differentiation into macrophages and the inflammasome was activated with LPS. The stimulated producer macrophages were placed into a co-culture as described earlier. When target THP-1 cells were co-cultured with macrophages that were pretreated with LPS, the expression of IL-1β and TNFα increased dramatically in target macrophages (Fig. 2D). However, expression of ncRILP in the stimulated producer cells completely blocked IL-1β and TNFα expression in target THP-1 cells. Similar increases in pro-inflammatory markers occurred in hepatocytes (Huh7.5 and HepG2) (Fig. 2E,F). The hepatocyte-derived pro-inflammatory markers HMGB1 and IL-33 increase when the cells were co-cultured with inflammatory producer macrophages. These increases were completely blocked in target hepatocytes when co-cultured with ncRILP-expressing inflammatory producer macrophages. Taken together, the data shows that the presence of a non-cleavable RILP in producer cells regulates the production of signals responsible for cell-to-cell transfer of inflammatory signals and suggests that blocking RILP cleavage dramatically inhibits pro-inflammatory cell–cell communication.

### RILP-regulation of cellular crosstalk in a model of animal-derived macrophages

**Fig. 3.**
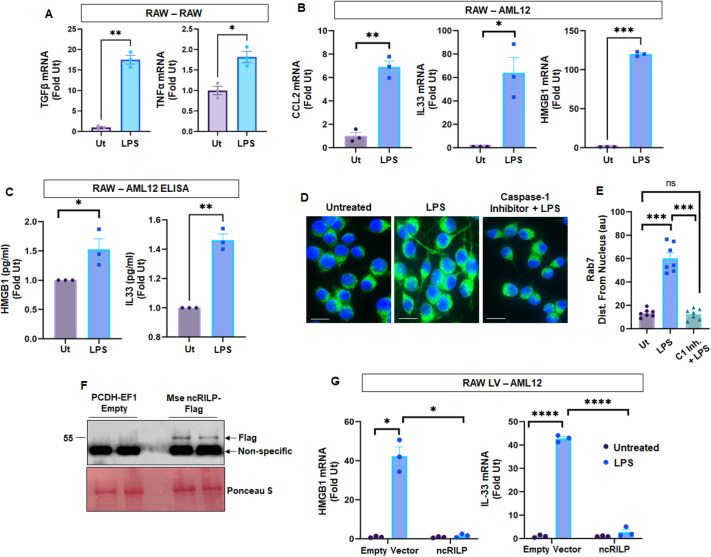
**RILP-mediated regulation of cellular crosstalk in mouse macrophages.** (A) RAW–RAW co-culture. Inflammatory markers increase in naïve RAW 264.7 cells when they are co-cultured with LPS-treated producer mouse macrophages. *n*=3. (B) Co-culture of LPS-treated RAW 264.7 cells with naïve AML12 hepatocytes showing increased expression of hepatocyte-specific cell injury markers within the target hepatocyte. *n*=3. (C) After co-culturing, AML12 target hepatocytes were collected, and the intracellular protein content was assessed by ELISA. Corresponding increases in HMGB1 and IL-33 are seen. *n*=3. (D) Caspase-1-mediated RILP cleavage in LPS-treated producer RAW 264.7 cells macrophages was confirmed by measuring the cellular distribution of Rab7. Scale bars: 15 µm. (E) The bar graphs represent an ImageJ analysis measuring the distance the Rab7-positive puncta are from the nucleus. *n=*a minimum of 30 cells from seven individual experiments. (F) Expression of ncRILP-Flag in RAW 264.7 producer cells showing two independent experiments. Western blot analysis for Flag resulted in a non-specific band at ∼43 kDa, which is present in all samples, regardless of transfection with empty vector (pCDH-EF1) or ncRILP–Flag. However, we also detected a band that only reacted with the anti-Flag antibody. This band is present in only the samples transfected with ncRILP–Flag. (G) Hepatocyte-derived pro-inflammatory markers HMGB1 and IL-33 increase in target AML12 cells when they are co-cultured with LPS-treated producer RAW 264.7 cells. These increases are completely blocked when ncRILP is expressed in the inflammatory macrophages. *n*=3. All data are shown as mean±s.e.m. *P≤0.05; **P≤0.01; ***P≤0.001; ****P≤0.0001; ns, not significant (two-tailed unpaired Student's *t*-test). Ut, untreated.

The data shows that RILP cleavage plays a critical role in the transfer of inflammatory mediators. Because cell–cell crosstalk is an important driver in the progression of several disease states, we wanted to assess the role of RILP cleavage in the promotion of inflammatory liver disease using a mouse model. To do this, we first confirmed that the RILP-mediated effects occur similarly in cells of mouse origin. RAW 264.7 macrophages were treated with LPS and co-cultured with naïve RAW 264.7. LPS treatment of the producer RAW 264.7 cells induced significant increases in both TGFβ (20-fold) and TNFα (2-fold) mRNA expression in naïve target macrophages (Fig. 3A). Similar increases in hepatocyte-derived pro-inflammatory markers were seen when RAW 264.7 macrophages were co-cultured with AML12 hepatocytes (Fig. 3B). After treatment with LPS, the mRNA of injury markers including IL-33, CCL2, and HMGB1 increased dramatically in target AML12 cells. Further analysis of target AML12 cell lysates by ELISA show corresponding increases in HMGB1 and IL-33 (Fig. 3C). We next assessed whether the inflammatory signal originating from the RAW 264.7 macrophages could be modulated by RILP manipulation. We first confirmed RILP cleavage by measuring Rab7 distribution. In control, untreated conditions, Rab7 localizes to the perinuclear region. After inflammasome activation with LPS, Rab7 redistributes towards the plasma membrane, indicative of RILP cleavage (Fig. 3D). Because RILP is a direct target of caspase-1 (Adams et al., 2018), we treated inflammatory macrophage with the caspase-1 inhibitor Z-YVAD-FMK. The addition of caspase-1 inhibitor to LPS-treated cells restored Rab7 distribution to the perinuclear region further confirming caspase-1-mediated RILP cleavage in RAW 264.7 macrophages (Fig. 3D,E). To determine the effect of blocking RILP cleavage on cell-to-cell crosstalk, producer RAW 264.7 macrophages were transfected with ncRILP (Fig. 3F), treated with LPS, and co-cultured with target AML12 hepatocytes. LPS treatment of producer RAW 264.7 cells induced the expression of HMGB1 and IL-33 in target hepatocytes. However, expression of ncRILP in producer macrophages had a protective effect on the target hepatocytes and dramatically inhibited target hepatocyte induction of pro-inflammatory genes (Fig. 3G).

**Fig. 4.**
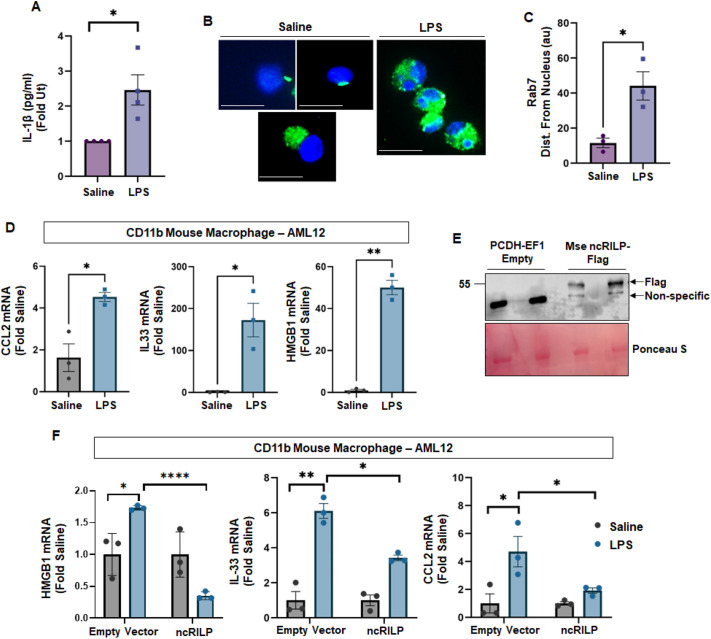
**RILP-mediated regulation of cellular crosstalk in an *ex vivo* model of animal-derived macrophages.** (A) C57BL/6 mice were injected with saline or LPS (0.5 mg/kg body weight). CD11b-enriched primary mouse macrophages were isolated and cultured. IL-1β secretion from the conditioned medium was measured by ELISA. There is a significant increase in the secretion of IL-1β from CD11b-enriched primary mouse macrophages that were isolated from LPS-injected mice. *n*=3. (B,C) CD11b-enriched mouse macrophages isolated from LPS-treated mice also show a significant redistribution of Rab7, thus confirming both inflammasome activation and RILP cleavage in this model. *n=*a minimum of 30 cells from seven individual experiments. Scale bars: 15 µm. (D) Co-culture of CD11b-enriched primary mouse macrophages and AML12 hepatocytes. CD11b-enriched cells isolated from the LPS-treated mice induce a significant expression of the injury markers CCL2, IL-33 and HMGB1 in target AML12 cells. *n*=3. (E) Expression of ncRILP–Flag in CD11b-enriched primary mouse macrophages. Figure shows two independent experiments. Western blot for Flag resulted in a non-specific band at ∼43 kDa, which is present in all samples, regardless of transfection with empty vector (pCDH-EF1) or ncRILP–Flag. We also detected a band that only reacted with the Flag antibody. This band is present in only the samples transfected with ncRILP–Flag. (F) CD11b-enriched primary macrophages were transfected with ncRILP and co-cultured with AML12 cells. Expression of ncRILP significantly decreased the inflammatory response in the hepatocyte. *n*=3. All data are shown as mean±s.e.m. *P≤0.05; **P≤0.01; ****P≤0.0001 (two-tailed unpaired Student's *t*-test).

We subsequently assessed this mechanism in cells derived from a mouse model of liver inflammation. C57BL/6 mice were injected with saline or LPS at a dose of 0.5 mg/kg of body weight. After 4 h, the liver was digested by perfusion with collagenase, and non-parenchymal cells (NPCs) were isolated. We removed neutrophils using anti-Ly6G microbeads and obtained a CD11b-enriched macrophage fraction by incubating the Ly6G flow-through with CD11b microbeads. The CD11b-enriched cells were then cultured, and the amount of secreted IL-1β was measured. Macrophage isolated from LPS-treated mice showed a significant increase in IL-1β secretion (Fig. 4A) as well as a redistribution in Rab7 (Fig. 4B,C) thus confirming both inflammasome activation and RILP cleavage in this model.

We next performed co-culture experiments using AML12 cells as the target hepatocytes. CD11b-enriched primary macrophages isolated from the LPS-treated mice induced a significant expression of the injury markers CCL2, IL-33 and HMGB1 in target AML12 cells (Fig. 4D). To determine whether the injury signal was transferred in a RILP-dependent manner, we transfected the CD11b-enriched primary macrophages with ncRILP (Fig. 4E) and performed a co-culture with AML12 cells. Expression of ncRILP significantly decreased the inflammatory response in the target hepatocytes (Fig. 4F). We noted that CD11b-enriched macrophages isolated from LPS-treated mice did not always produce similarly robust responses in target cells. Inflammatory responses are temporal, and it is possible that we missed the more robust responses due to the timing of lentiviral transduction and/or the inherent variably of liver-derived macrophages. Nonetheless, the data show that RILP cleavage plays a substantial role in the transfer of inflammatory mediators in primary cells derived from a mouse model of liver inflammation.

### Effects of ncRILP on the components of the inflammatory response

**Fig. 5.**
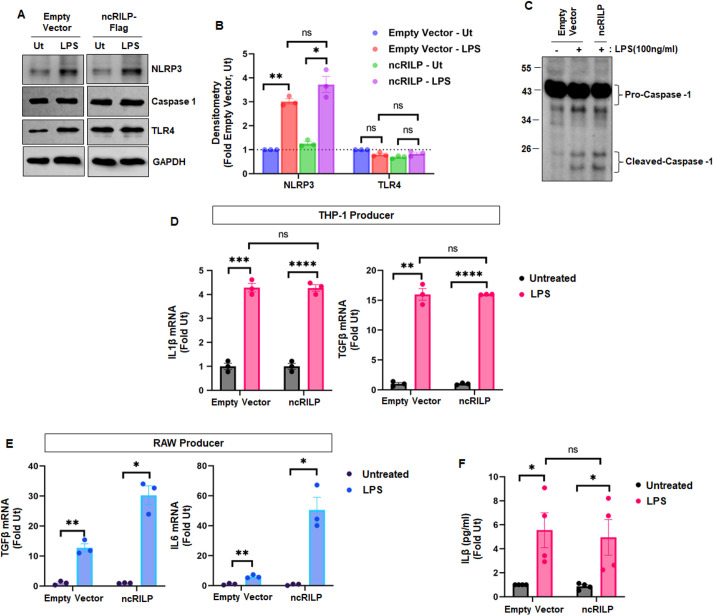
**Effects of ncRILP on the components of the inflammatory response.** (A) The expression of ncRILP had no effect on the levels of inflammasome components (NLRP3) or the LPS receptor (TLR4). (B) Densitometry analysis of A. *n*=3. (C) ncRILP expression did not block the ability of the producer cell to induce caspase-1 activation. Image representative of three repeats. (D,E) qPCR analysis of cells that were transduced with empty vector or ncRILP lentivirus and treated with LPS. The expression of ncRILP does not affect the expression or induction of inflammatory genes in THP-1 cells or RAW 264.7 cells. *n*=3. (F) ncRILP does not affect the secretion of inflammatory cytokines. THP-1 cells were treated with LPS and the presence of IL-1β was measured in the medium by ELISA. *n*=4. All data are shown as mean±s.e.m. *P≤0.05; **P≤0.01; ***P≤0.001; ****P≤0.0001; ns, not significant (two-tailed unpaired Student's *t*-test). Ut, untreated.

The above data show that the RILP cleavage status of macrophages influences hepatocellular markers of injury. We further show that expressing ncRILP in producer macrophages alters the inflammatory cell–cell communication in target cells. Next, we examined mechanism by which ncRILP blocks the transfer of an injury signal to target cells as well as the source of the inflammatory signal. First, we assessed whether ncRILP expression directly altered the expression of inflammasome components in producer cells. As expected, treatment with LPS increased NLRP3 protein levels in producer macrophages, consistent with inflammasome activation (Fig. 5A,B). The expression of ncRILP had no effect on NLRP3 levels or inflammasome activation. Furthermore, ncRILP did not alter the expression of TLR4, the LPS receptor, in producer macrophages. Finally, ncRILP did not block the ability of the producer cell to induce caspase-1 activation (Fig. 5C).

Next, we examined whether ncRILP affects the ability of macrophages to respond to an inflammatory stimulus. We treated empty vector-transfected and ncRILP-expressing producer THP-1 cells with LPS and measured total cellular RNA. No difference was observed in the expression of IL-1β or TGFβ between the empty vector and ncRILP group (Fig. 5D). Interestingly, ncRILP expression increased IL-6 and TGFβ responses to LPS (Fig. 5E). Finally, we looked at the effect of ncRILP expression on inflammatory cytokine secretion. LPS treatment of producer THP-1 cells increased IL-1β secretion ∼4-fold. ncRILP expression had no effect on IL-1β secretion (Fig. 5F). Altogether, these data show that the mechanism by which ncRILP blocks the transfer of an inflammatory signal to target hepatocytes is not due to the downregulation of inflammatory cytokine production or secretion in the producer cells.

### EVs are the source of the inflammatory macrophage-derived signal

**Fig. 6.**
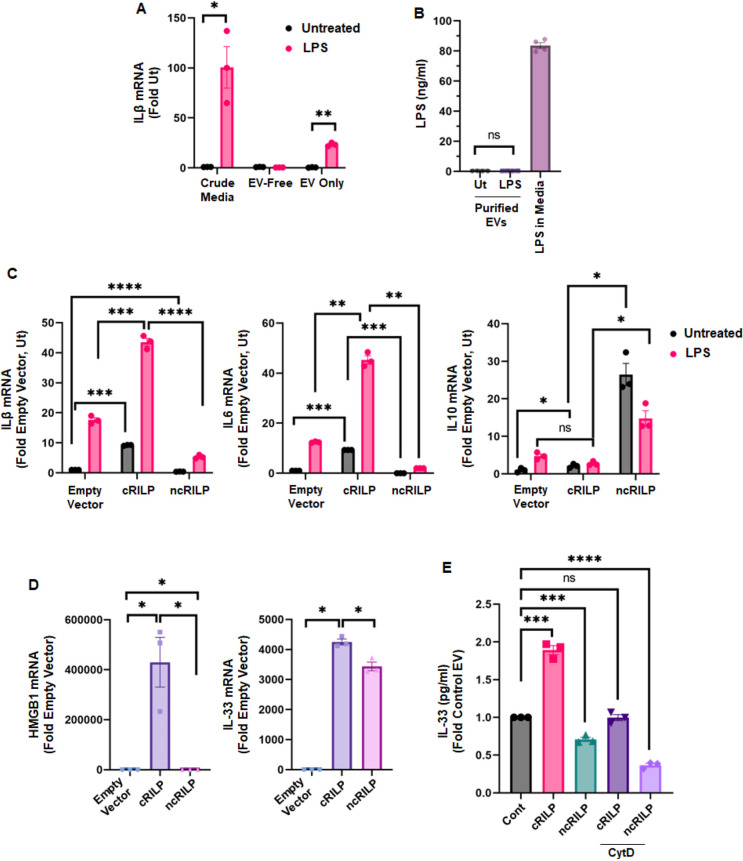
**EVs are the source of the inflammatory macrophage-derived signal.** (A) qPCR analysis of THP-1 cells that were treated with crude conditioned medium, an EV-free soluble fraction or purified EVs (5 µg) for 24 h. Only treatment with crude medium and purified EVs induced an inflammatory response. *n*=3. (B) No LPS was detected in the EV preparations, assuring that any EV-mediated effect seen was not due to the carry-over of LPS. The amount of LPS in medium alone serves as a positive control. *n*=4. (C,D) EVs derived from ncRILP-expressing cells are protective. THP-1 (C) or Huh7.5 (D) cells were incubated with cRILP or ncRILP EVs and treated with LPS. qPCR analysis shows that treatment with cRILP EVs increases levels of several inflammatory markers (IL-1β, IL-6, HMGB1 and IL-33). Treatment with ncRILP EVs protects against LPS-mediated inflammation and significantly reduces the expression of inflammatory markers. Note an increase in the anti-inflammatory marker IL10 with ncRILP. *n*=3. (E) The EV-mediated crosstalk model was further validated in Huh-7.5 cells by using the EV uptake inhibitor (cytochalasin D, CytD). Cells were treated with CytD (10 μM for 30 min) and then incubated with THP-1 derived EVs. Cell lysates were collected, and IL-33 ELISA was performed. In presence of inhibitor, cRILP EVs no longer induce IL-33 expression in Huh-7.5 cells. These results confirm that EVs are the key regulators of cellular crosstalk. *n*=3. All data are shown as mean±s.e.m. *P≤0.05; **P≤0.01; ***P≤0.001; ****P≤0.0001; ns, not significant (two-tailed unpaired Student's *t*-test). Ut, untreated.

Previous work from our lab has shown that RILP cleavage reroutes cellular vesicle trafficking toward the cell surface. This results in enhanced EV secretion (Wozniak et al., 2020). We further showed that formation of the cleaved form of RILP (cRILP) induces unique exosomal cargo loading, leading to the selective enrichment of specific pro-inflammatory cargo. Inhibiting RILP cleavage via the expression of ncRILP might thus abrogate the proinflammatory actions generated by cRILP while specifically facilitating an anti-inflammatory response. Because ncRILP expression did not alter the secretion of pro-inflammatory cytokines such as IL-1β, we hypothesized that inflammatory macrophages transfer an injury signal to target cells via EVs. To test this, cell culture supernatants were collected from control and LPS-treated THP-1 cells and subjected to differential centrifugation to separate the EV-free soluble fraction from the EV pellet (Fig. S3A) (Thery et al., 2006). Confirming previous analyses performed by our lab (Wozniak et al., 2020), western blot analysis showed that the EV pellets contain known exosomal markers (CD63 and flotillin) but do not contain the microvesicle marker annexin A1 or components of other organelles, including the Golgi (Fig. S3B). Naïve THP-1 cells were then treated with either the crude conditioned medium, the EV-free soluble fraction, or the purified EVs (5 µg) derived from the conditioned medium for 24 h before RNA isolation. Treatment with crude conditioned medium from LPS-treated THP-1 cells resulted in a dramatic increase in the pro-inflammatory marker IL-1β whereas the EV-free soluble fraction had no effect (Fig. 6A). In contrast, treatment of THP-1 cells with purified EVs resulted in a 30-fold increase in IL-1β. To assure that any EV-mediated effect seen was not due to the carry-over of LPS, we measured LPS in the purified EV preparations. As a control, we incubated culture medium with 100 ng/ml LPS and after 24 h, measured the amount of LPS remaining in the medium. No LPS was detected in the EV preparations (Fig. 6B). Together, these data show that inflammatory cells transfer their injury signal via EVs.

Because cRILP and ncRILP differentially regulate exosomal cargo (Wozniak et al., 2020), we hypothesized that the ability of ncRILP to protect against an inflammatory response was due to the differences in cargo within the EV. To assess this, EVs were purified from cRILP- or ncRILP-expressing THP-1 cells and used to inoculate naïve THP-1 cells. Interestingly, treatment with cRILP EVs alone induced the expression of IL-1β and IL-6 yet did not increase the levels of the anti-inflammatory cytokine IL-10 (Fig. 6C). This confirms previous results showing that cRILP expression in producer cells can enrich for pro-inflammatory cargo. The combination of cRILP EVs and LPS had an additive effect, causing considerable increases in two inflammatory genes, whereas this had little to no effect on the anti-inflammatory marker IL-10 (Fig. 6C, right panel). In contrast, EVs isolated from ncRILP-expressing cells, protected against LPS-mediated inflammation, significantly reducing the expression of inflammatory markers after the cells were treated with LPS. Interestingly, treatment with ncRILP-derived EVs alone increased IL-10 expression.

We next examined whether macrophage-derived ncRILP EVs could protect hepatocytes from an injury signal. Because hepatocytes do not readily respond to LPS, we examined the effect of EV treatment alone. Huh-7.5 cells were treated with EVs purified from cRILP- or ncRILP-expressing THP-1 cells as above. cRILP EVs induced the expression of the inflammatory markers HMGB1 and IL-33 in the hepatocyte. Notably, when treated with EVs isolated from ncRILP-expressing THP1 cells, a reduction in these markers was seen (Fig. 6D).

The EV-mediated crosstalk model was further validated in Huh-7.5 cells by using the EV uptake inhibitor cytochalasin D (Bastos-Amador et al., 2012; Catalano and O'Driscoll, 2020). Huh-7.5 cells were treated with cytochalasin D (10 µM for 30 min) and treated with EVs purified from THP-1 cells. Huh-7.5 cell lysates were then collected and subjected to ELISA for IL-33. In the presence of the EV uptake inhibitor, cRILP EVs no longer induced IL-33 expression in Huh-7.5 cells (Fig. 6E). Together, these data suggest that not only are EVs the source of macrophage-induced injury signals during inflammation but that blocking RILP cleavage differentially shifts the inflammatory phenotype of the EV.

## DISCUSSION

Cell–cell communication is a fundamental feature of the hepatic microenvironment, and a main form of cellular communication occurs through the trafficking of vesicles between intracellular compartments as well as between the cells themselves. This signaling plays critical roles in disease progression as well as resolution; however, the mechanisms by which this occurs are not fully understood. In this study, we establish a direct link between the macrophage secretome and hepatocyte function, and show that we can reprogram inflammatory macrophage EV production to suppress the transmission of an injury signal. Using a co-culture system, we show that LPS-treated macrophages transfer an inflammatory signal to both naïve macrophages and hepatocytes. We systematically analyzed the extracellular content to determine the source of the inflammatory signal. Using differential centrifugation to separate the soluble fraction from the EV-associated material, we found that EVs isolated from LPS-treated macrophages were sufficient to increase the expression of inflammatory markers in target cells.

Inflammasome activation is regulated by a multi-step process. The priming step (signal 1) is provided by microbial or endogenous molecules that induce the expression of inflammasome components. The activation step (signal 2) is triggered by a shift in ion homeostasis, thereby promoting inflammasome assembly and caspase-1-mediated cleavage of target proteins (He et al., 2016; Latz et al., 2013). However, LPS alone is also able to activate the inflammasome in several circumstances (Dalby, 2018; Monguió-Tortajada et al., 2018). In this study, we chose to use a long-term treatment with a lower dose of LPS (100 ng/ml) and show that this longer period is sufficient to fully activate the inflammasome without the addition of ATP.

In previous work, we have shown that intracellular trafficking pathways are hijacked and/or altered during inflammation, only to exacerbate the injury (Wozniak et al., 2020). This occurs via inflammasome-induced cleavage of the Rab7 adaptor protein RILP. RILP cleavage repositions cellular vesicles, leading to an enhancement of plasma membrane fusion events. It also accounts for a burst of secretory events including a dramatic increase in the release of EVs. In this study, we further connected LPS-mediated inflammatory EV secretion to the cleavage of the trafficking adaptor protein RILP and show that EVs isolated from cRILP-expressing macrophages were themselves able to produce these effects. We also show that inhibiting RILP cleavage via the expression of the non-cleavable form of RILP, ncRILP, imparts a protective effect on target cells, and our data suggests this effect is mediated via EV transfer. We confirmed that ncRILP does not alter the ability of the producer macrophages to respond to inflammatory stimuli as there was no change in TLR4 expression or the expression of inflammasome components including caspase-1 or NLRP3. Furthermore, the secretion of inflammatory cytokines was not affected by ncRILP expression. The finding that ncRILP expression did not alter producer cell inflammatory responses or general EV-mediated secretory processes but did influence downstream signaling in targets cells suggests that ncRILP functions to influence EV cargo loading.

Intercellular communication is mediated by direct cell–cell contact, soluble factors, including cytokines and growth factors, and EVs. These mechanisms work in combination to maintain homeostasis by responding appropriately to conditions of stress. Equally, mis-regulation of any of these mechanisms can promote altered physiology leading to disease. In this study, we separated the EV-mediated effects from those initiated by soluble cytokines thereby giving a broader insight into the mechanisms that both promote and resolve inflammation. EVs have received a lot of attention as not only biomarkers but also as a manipulatable entity for therapy (Masyuk et al., 2013). They play critical roles in cell–cell communication during both healthy and diseased states. In healthy states, they preserve and restore cellular homeostasis. In diseased states, they exacerbate injury by initiating numerous inflammatory signaling cascades. The latter is particularly true in nonalcoholic steatohepatitis (NASH) where an increase in the number of circulating EVs correlates to disease progression (Kakazu et al., 2016; Tsuchiya et al., 2022). However, the mechanism by which inflammation alters the biogenesis and disease-associated changes in exosomal cargoes is not fully known. We show here that EVs isolated from ncRILP-expressing cells are intrinsically anti-inflammatory and in previous work we have shown that the RILP cleavage status differentially alters the miRNA selectivity of EV cargo loading. The cleaved form of RILP enriches for pro-inflammatory miRNAs within the EV. Blocking RILP cleavage specifically enhances for anti-inflammatory miRNAs within the EV while simultaneously suppressing exosomal miRNAs that are involved in pro-inflammatory processes (Wozniak et al., 2020). The data therefore suggest that ncRILP changes the cargo present within the EVs and this cargo has a protective effect on target cells. Further support for this was obtained by assessing the effect of treating hepatocytes with purified EVs. EVs derived from ncRILP-expressing macrophages blocked LPS-induced inflammatory responses in hepatocytes.

We noted that although ncRILP did not alter the production or secretion of inflammatory cytokines in response to LPS, it did induce the expression of IL-6 and TGFβ in producer macrophages after treatment. It is not uncommon for cytokines to play dual roles in the progression of inflammatory disease and tissue homeostasis. For example, TGFβ plays a different role in lean mice whereby the macrophages maintain an anti-inflammatory phenotype. In obese mice this phenotype is lost, and the macrophages release pro-inflammatory cytokines which ultimately contribute to and promote the inflammatory environment (Kawanishi et al., 2010; Wu et al., 2011). Although we did not specifically determine whether these cytokines played a role in the ability of ncRILP to impart protective effects on target cells, we do show that treatment with ncRILP-derived EVs alone can mediate this protective effect and previous work from our lab has shown that cRILP and ncRILP differentially interact with components of EV biogenesis and loading machinery to regulate cargo loading. It is possible that the cytokines themselves are loaded into the EVs. This has been shown in human mast cells where treatment with IL1 pushes IL6 into secreted vesicles (Barnes and Somerville, 2020; Metcalfe et al., 1997). Therefore, it is possible that ncRILP functions via multiple mechanisms, both by inducing the expression of specific cytokines and influencing their loading into the EV.

In conclusion, it is well documented that the inflammatory macrophage secretome can alter the fate and function of target cells, thereby contributing to disease progression. This work highlights a previously unrecognized mechanism through which this occurs. We show that cleavage status of RILP programs EV biogenesis has profound effects on the cell–cell transmission of an inflammatory state. cRILP-derived EVs themselves promote inflammation in target cells whereas blocking RILP cleavage via ncRILP expression completely shifts exosomal programing and imparts a protective effect on target cells. This means there is potential that RILP cleavage could be exogenously manipulated so as to direct EV biogenesis for therapeutic benefit.

## MATERIALS AND METHODS

### General materials and antibodies

General materials were purchased from Sigma-Aldrich or VWR. RPMI, DMEM, FBS Opti-MEM, and Lipofectamine 3000 were purchased from Thermo Fisher Scientific. LPS was from Enzo (ALX-581-013-L002). Protease inhibitor cocktail (P8340; Sigma-Aldrich) was used at 1:100 dilution. Antibodies against caspase-1 (Abcam ab207802; 1:1000), TLR4 (Abcam ab13556; 1:1000), NLRP3 (Cell Signaling Technology, 15101 s; 1:1000), Flag M2 (Sigma, F1804) and GAPDH (Santa Cruz Biotchnology, FL-335; 1:500) were used in this study. Caspase-1 inhibitor VI (Z-YVAD-FMK, Sigma 218746) was used at 100 µM. Antibodies were verified by the individual companies. Cytochalasin D (Sigma, C2618) was used at 10 µM. These concentrations were not associated with toxicity.

### Plasmids

All pLVX-IRES-mCherry-based RILP plasmids including vectors for HA–RILP–Flag, HA–ncRILP–Flag and cRILP–Flag have been described previously (Wozniak et al., 2020, 2016). Mouse RILP (pUCIDT-AMP: HA-RILP-Flag, accession NM_001029938.2) was generated by Integrated DNA Technologies. The region encoding HA-mseRILP-Flag was excised and cloned into pCDH-EF1 (Addgene plasmid #72266, deposited by Kazuhiro Oka). Mouse-ncRILP was generated using Q5 Site-Directed Mutagenesis Kit (NEB) by mutating amino acids 80–84 within the RILP sequence to alanine residues to give the plasmid HA–Mse-ncRILP–Flag. All sequences were confirmed by DNA sequencing analysis. Plasmids are available in Addgene (plasmids #102425 and #102424) or upon request.

### Cell culture and transfection

Huh-7.5 cells were obtained from Charles Rice (Rockefeller University, New York, NY) and cultured in DMEM containing 4.5 g/l glucose, L-glutamine and sodium pyruvate, 10% FBS and 1% nonessential amino acids. THP-1 human monocyte cells were purchased from ATCC (TIB-202) and cultured in RPMI 1640 containing 10% FBS. RAW 264.7 cells were purchased from ATCC (TIB-71) and cultured in DMEM supplemented with 10% FBS. AML12 cells were from ATCC (CRL-2254) and cultured in DMEM/F12, supplemented with 10% FBS, 10 µg/ml insulin, 5.5 µg/ml transferrin, 5 ng/ml selenium and 40 ng/ml dexamethasone. All cells were maintained at 37°C and 5% CO_2_. All cell lines had been authenticated and are routinely tested for contamination using the MycoAlert Mycoplasma Detection Kit (Thermo Fisher Scientific, NC9922140). Transfections were performed using Lipofectamine 3000 according to the manufacturer's instructions.

### Lentivirus production and transduction

For lentivirus production, 293FT cells were plated in 10 cm^2^ dishes and transfected with 1.95 μg psPax2, 650 ng pMDG.2, and 2.6 μg of either the mCherry-based empty vector, cRILP–Flag or ncRILP–Flag plasmids. 15.6 μl of X-tremeGENE HP transfection reagent (Roche) was used. The next day, the medium was replaced with antibiotic-free complete medium (DMEM supplemented with 10% FBS, 1× MEM nonessential amino acids, 6 mM L-glutamine and 1 mM sodium pyruvate). Supernatants were collected daily for the next 72 h.

For transduction, THP-1 cells were plated at a concentration of 1×10^6^ cells per ml in 150 mm^2^ dishes. Lentivirus (10 ml) was incubated at room temperature with polybrene (8 μg/ml) for 15 min. The virus was then added to THP-1 cells and incubated overnight at 37°C and 5% CO_2_. The next day, PMA (100 ng/ml) was added to differentiate THP-1 into macrophage-like cells. After 4 h, the cells were washed, and the medium was replaced with complete medium. After a 48-h rest period, the cells were washed in PBS and the medium was replaced with EV-free complete medium.

### EV isolation and analysis

THP-1 cells (10^6^ cells per ml in 150 mm^2^ dishes) were cultured in 15 ml complete medium supplemented with 10% FBS (depleted of bovine EVs by overnight centrifugation at 100,000 ***g***). EVs were isolated from cell culture supernatants via a series of differential ultracentrifugations using a Beckman SW32Ti Swinging Bucket Rotor (Thery et al., 2006). The final EV pellets were resuspended in PBS. EV protein concentration was measured by standard Bradford dye assay as undertaken previously (Wozniak et al., 2020). Western blot characterization of the EV preparation has been reported by us previously (Wozniak et al., 2020) and was performed in this study.

### Co-culture system

For co-culture experiments, producer cells (THP-1 or RAW 264.7) were seeded in the upper chamber of a transwell plate (Corning 3428) on day 1, according to manufacturer's instruction. THP-1 cells were also treated with PMA (100 ng/ml) overnight, to differentiate them into M0 macrophages. On day 2, the cells were washed with PBS and the medium was replaced with complete medium. On day 3, the macrophages were treated with LPS (100 ng/ml) for 24 h. On day 4, the macrophages were washed with PBS to remove any traces of LPS. The upper transwell containing the macrophages was then transferred to another well of a 6-well plate containing the target cells (Huh-7.5, HepG2 or AML12). We used a 1:2 ratio of macrophage versus target cells for each co-culture experiment. Complete medium was added in both upper and lower chamber. The co-culture proceeded for at least 6 h. At the desired time points, cells were collected for RNA isolation and protein.

### RNA isolation and real-time PCR

RNA was extracted from producer and target cells using TRI reagent (Sigma-Aldrich) according to the manufacturer's instructions. cDNA was generated with the random primer method using the RNA reverse transcription kit (Applied Biosystems). Quantitative RT-PCR was performed in a CFX96 real-time system (Bio-Rad) using specific sense and antisense primers in 20 μl reactions (primers are listed in Table S1). All Cq values were normalized to their own GAPDH value.

### Western blotting and ELISA

Whole-cell lysates were prepared from cells lysed in 1× RIPA buffer (20 mM Tris-HCl pH 7.4, 150 mM NaCl, 1 mM EDTA, 1 mM EGTA, 1% Triton X-100, 2.5 mM Na_4_P_2_O_7_ and protease inhibitors). Cell lysates were separated by SDS-PAGE and transferred to polyvinylidene difluoride membranes (Millipore). Membranes were blocked with 5% milk and 0.1% Tween-20 in TBS for 1 h at room temperature. Membranes were incubated with the appropriate primary antibodies overnight at 4°C. Finally, membranes were incubated with HRP-conjugated secondary antibodies for 1 h at room temperature and detected using the Pierce ECL Western blotting substrate kit (Thermo Fisher Scientific) and the Odyssey Fc, Dual-Mode Imaging system (Li-COR). The detection of IL-1β in the culture medium was undertaken using the human IL-1β Simple Step ELISA kit from Abcam (ab100562) and mouse IL-1β Simple Step ELISA kit from Abcam (ab197742) according to the manufacturer's instructions. The detection of IL-33 from cell lysates was undertaken using the human IL-33 Simple Step ELISA kit from Abcam (ab223865) and mouse IL-33 Simple Step ELISA kit from Abcam (ab213475) according to the manufacturer's instructions. Human and mouse HMGB1 was detected from cell lysates using human HMGB1 (Novus Bio, NBP2-62766) and mouse HMGB1 (Novus Bio, NBP2-62767) ELISA kits.

### Immunofluorescence

Cells grown on coverslips were fixed in 4% PFA at room temperature for 30 min, permeabilized with 0.1% Triton X-100 for 5 min at room temperature and incubated in immunofluorescence buffer (1% BSA and 2.5 mM EDTA in PBS) for 1 h at room temperature. After washing in PBS, the cells were incubated in primary antibody diluted in immunofluorescence buffer containing 0.1% Triton X-100, overnight at 4°C. Primary antibodies used were rabbit anti-Flag antibody (1 µg/ml, F7425; Sigma-Aldrich) and Rab7 D95F2 (1:200, Cell Signaling Technology 9367). After washing with PBS, the coverslips were incubated with Alexa Fluor-conjugated goat secondary antibody (1:1000) containing DAPI (1 µg/ml) for 1 h in the dark at room temperature and then mounted in FluorSave Reagent (Sigma, 345789). Images were acquired by using an Eclipse Ti microscope (Nikon Americas) and analyzed using ImageJ2 (Rueden et al., 2017; Schindelin et al., 2012).

### Endotoxin assay

EVs were isolated from control, untreated and LPS-treated macrophages by differential centrifugation as stated previously (see EV isolation and analysis and Thery et al., 2006). LPS levels were quantified in the EV preparations using the Pierce Chromogenic Endotoxin Quant Kit (A39552S) according to the manufacturer's instructions.

### Liver perfusion and isolation of CD11b-positive macrophages

C57/B6 wild-type chow male mice were used for this study. Mice were injected intraperitoneally (i.p.) with either saline or LPS at a dose of 0.5 mg/kg body weight. After 4 h, liver perfusion was performed, and non-parenchymal cells (NPCs) were isolated. Mice were anaesthetized through i.p. injection of xylazine and ketamine solution at a dose of 0.01 ml/g body weight. The liver was perfused, and NPCs were collected (Charni-Natan and Goldstein, 2020; Troutman et al., 2021). To remove neutrophils, the NPCs were incubated with mouse Ly-6G beads (Miltenyi Biotec 130-120-337) for 15 min at 4°C and passed through a magnetic column (Miltenyi Biotec 130-042-041). The flow-through was collected and incubated with CD11b beads (Miltenyi Biotec 130-049-601) to isolate all macrophages including resident and infiltrating. The isolated macrophages were then plated in transwell for co-culture experiments. All animal studies are in compliance with the University of Kansas Medical Center IACUC guidelines.

### Statistics

All experimental results represent observations from at least three biological replicates. All data are presented as mean±s.e.m. A two-tailed unpaired Student's *t*-test was used (GraphPad Prism) for statistical analyses. *P*≤0.05 was considered significant, and is indicated as *P≤0.05, **P≤0.01, ***P≤0.001 and ****P≤0.0001.

## Supplementary Material

10.1242/joces.260691_sup1 Supplementary information
