# Supplementary material for: Inflammatory macrophage to hepatocyte signals can be prevented by extracellular vesicle reprogramming
Source: J Cell Sci. 2023 May 12;136(9):jcs260691. doi: 10.1242/jcs.260691 (PMC10184766; doi:10.1242/jcs.260691)
Supplement: Supplementary information [file joces-136-260691-s1.pdf]

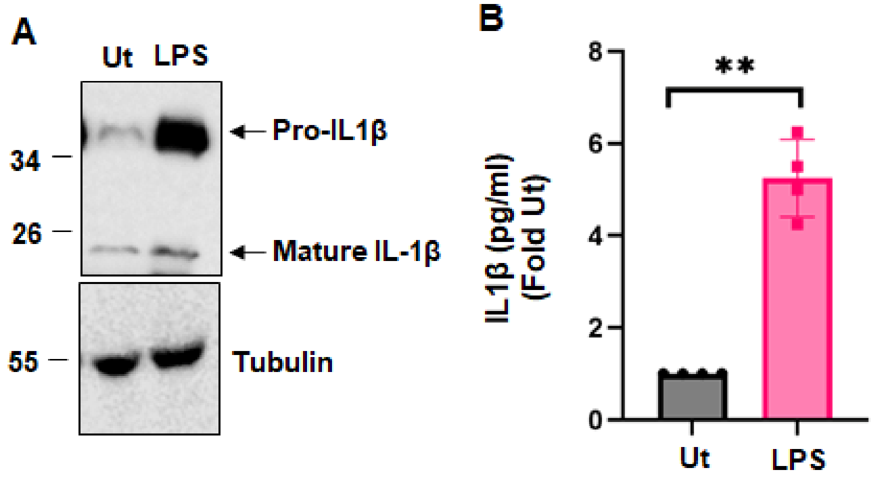

Fig. S1. Inflammasome activation in THP-1 producer cells. (A) Western blot analysis of producer THP-1 cells treated with 100ng/ml LPS for 24hr. The amount of mature IL-1β is increased after LPS treatment. **(B)** IL-1β ELISA of conditioned media isolated from producer THP-1 cells treated with LPS. Data are shown as mean ± SEM for $n = 4$.

Journal of Cell Science • Supplementary information

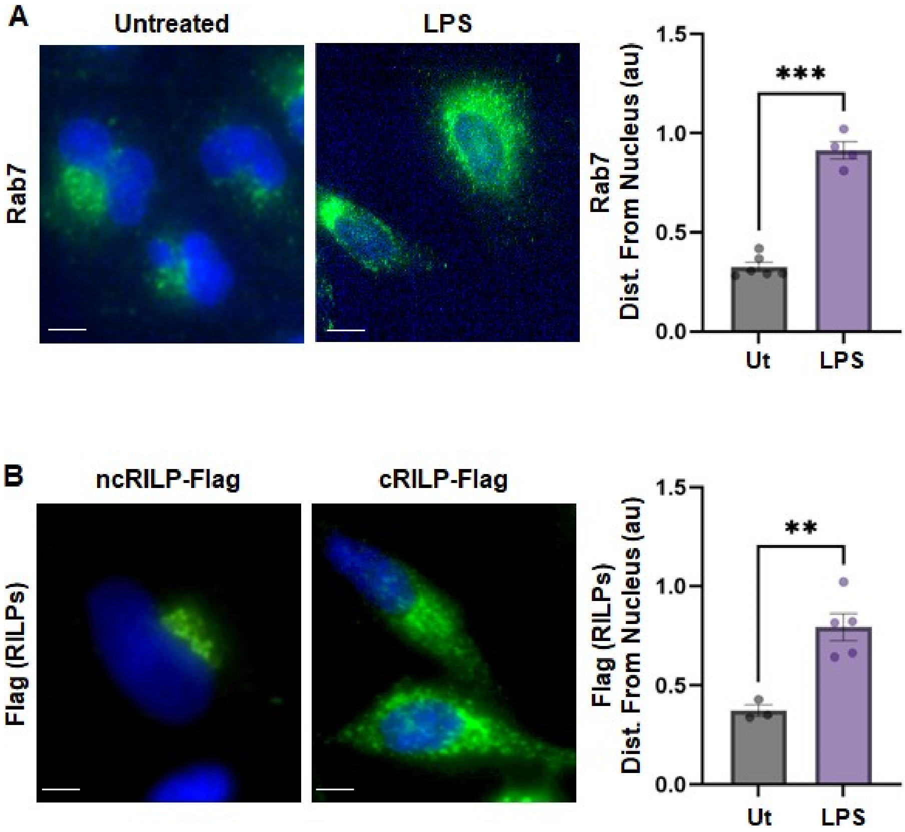

Fig. S2. Localization of Rab7 as a surrogate for RILP cleavage. (A) In HMC3 microglia cells, Rab7 serves as a marker of RILP cleavage in immunofluorescence. At baseline, Rab7 localizes at the perinuclear region, near the mitotic center. This localization mirrors that of non-cleavable RILP **(B, ncRILP).** After RILP cleavage via inflammasome activation with LPS, Rab7 redistributes throughout the cellular periphery and extends to the plasma membrane. This localization is similar to that seen when cleaved RILP is overexpressed **(B, cRILP).** The bar graphs represent an ImageJ analysis measuring the distance the $Rab7^{+ve}$ or $RILP^{+ve}$ puncta are from the nucleus. Data are shown as mean ± SEM; *, $P \leq 0.05$ for n = a minimum of 15 cells from 3-5 individual experiments. Scale bar: 10µm.

Journal of Cell Science • Supplementary information

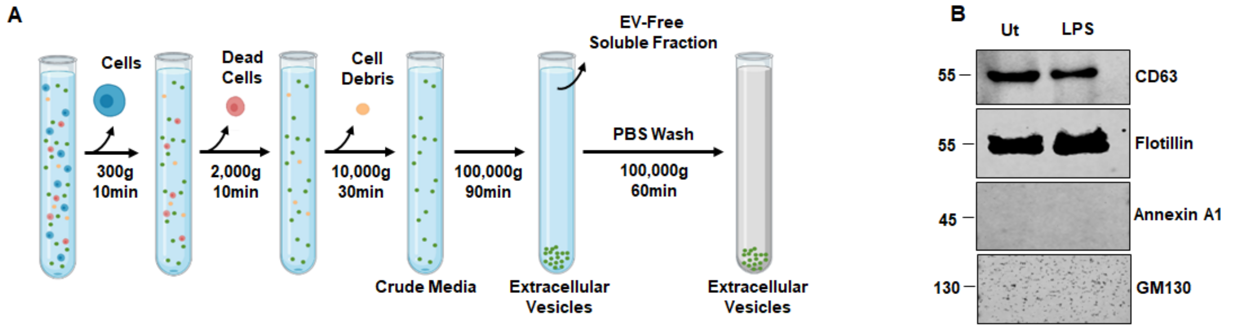

Fig. S3. (A) S3. (A) Cell culture superna Cell culture supernattants were coants were collecllectted froed from controm control and LPS-treal and LPS-treated THP-1 cellsted THP-1 cells and subjecand subjecteted to difd to difffererenentiatial cenl centrifutrifuggation to seation to separate the Eparate the EVV-free so-free solublublle fe frraction faction frroom the EVm the EV pellepellet.t. **(B) (B)** Western b Western blloott analysis sh analysis showed that the owed that the EV pellets contain known exosomal markersEV pellets contain known exosomal markers (CD63 and Flotillin(CD63 and Flotillin) bu) but do not do not cont conttainain the micr the microvesicle marker annexovesicle marker annexiin A1 or con A1 or componentsmponents of of other orgother organeanelles includlles includining the Golgi.g the Golgi.

Journal of Cell Science • Supplementary information

Table S1. Primers used in this study.

**Primer Name**	**Primer Sequence (5'-3')**
hIL-1β Forward	CATGGGATAACGAGGCTTATGT
hIL-1β Reverse	CCCAAGGCCACAGGTATTT
hIL6_Forward	ACTCACCTCTTCAGAACGAATTG
hIL6_Reverse	CCATCTTTGGAAGGTTCAGGTTG
hIL33_Forward	GTGACGGTGTTGATGGTAAGAT
hIL33_Reverse	AGCTCCACAGAGTGTTCCTTG
hCRP Forward	AGACATGTCGAGGAAGGCTTTT
hCRP Reverse	TCGAGGACAGTTCCGTGTAGAA
hHMGB-1-Forward	GGACAAGGCCCGTTATGAAA
hHMGB-1-Reverse	GCAGAAGAGGAAGAAGGCCGAA
hCCL2_Forward	ATGAAAGTCTCTGCCGCCCTTCT
hCCL2_Reverse	TGAGTGTTCAAGTCTTCGGAGTT
hTNFα_Forward	TCTTCTCGAACCCCGAGTGA
hTNFα_Reverse	CCTCTGATGGCACCACCAG
hIL10_Forward	TCAAGGCGCATGTGAACTCC
hIL10_Reverse	GATGTCAAACTCACTCATGGCT
hTGFβ-1forward	TATCGCCAGGAATTGTTGCTG
hTGFβ-1 reverse	CAATTCCTGGCGATACCTCAG
hGAPDH-F	GAAGGTGAAGGTCGGAGTC
hGAPDH-R	GAAGATGGTGATGGGATTTC
mCRP Forward	GAACTTTCAGCCGAATACATCTTTT
mCRP Reverse	CCTTCCTCGACATGTCTGTCT
mIL-1β-Forward	TCGCTCAGGGTCACAAGAAA
mIL-1β-Reverse	CATCAGAGGCAAGGAGGAAAAC
mTGF-β-Forward	GTGTGGAGCAACATGTGGAACTCTA
mTGF-β-Reverse	TTGGTTCAGCCACTGCCGTA
mIL-10-Forward	GGTTGCCAAGCCTTATCGGA
mIL-10-Reverse	ACCTGCTCCACTGCCTTGCT
mTNFα-Forward	AGGCTCTGGAGAACAGCACAT
mTNFα-Reverse	TGGCTTCTCTTCCTGCACCAAA
mArg1-Forward	CTCCAAGCCAAAGTCCTTAGAG
mArg1-Reverse	AGGAGCTGTCATTAGGGACATC
mCCL2-Forward	GTTGGCTCAGCCAGATGCA
mCCL2-Reverse	AGCCTACTCATTGGGATCATCTTG
mHMGB-1-Forward	GGCGAGCATCCTGGCTTATC
mHMGB-1-Reverse	GGCTGCTTGTCATCTGCTG
mIL6_Forward	TAGTCCTTCCTACCCCAATTTCC
mIL6_Reverse	TTGGTCCTTAGCCACTCCTTC
mIL33_Forward	TCCAACTCCAAGATTTCCCCG
mIL33_Reverse	CATGCAGTAGACATGGCAGAA
mGAPDH-6-Forward	CGTCCCGTAGACAAAATGGT
mGAPDH-6-Reverse	TTGAGGTCAATGAAGGGGTC

Journal of Cell Science • Supplementary information
