# Supplementary material for: Inflammatory macrophage to hepatocyte signals can be prevented by extracellular vesicle reprogramming
Source: J Cell Sci. 2023 May 12;136(9):jcs260691. doi: 10.1242/jcs.260691 (PMC10184766; doi:10.1242/jcs.260691)
Supplement: Supplementary file 1 [file joces-136-260691_review_history.pdf]

Journal of Cell Science

# Inflammatory macrophage to hepatocyte signals can be prevented by extracellular vesicle reprogramming

Priyanka Ghosh, Kyo Sasaki, Isabel Aranzazu Pulido Ruiz, Kayla E. King, Steven A. Weinman and Ann L. Wozniak
DOI: 10.1242/jcs.260691

**Editor**: Daniel Billadeau

**Review timeline**
Original submission: 7 October 2022
Editorial decision: 15 November 2022
First revision received: 13 March 2023
Accepted: 5 April 2023

---

**Original submission**

<u>First decision letter</u>

MS ID#: JOCES/2022/260691

MS TITLE: Inflammatory macrophage to hepatocyte signals can be prevented by exosome cargo reprogramming

AUTHORS: Priyanka Ghosh, Kyo Sasaki, Kayla E King, Steven A Weinman, and Ann L Wozniak
ARTICLE TYPE: Research Article

We have now reached a decision on the above manuscript.

To see the reviewers' reports and a copy of this decision letter, please go to: https://submit-jcs.biologists.org and click on the 'Manuscripts with Decisions' queue in the Author Area. (Corresponding author only has access to reviews.)

As you will see, all three reviewers raise a number of substantial criticisms that prevent me from accepting the paper at this stage. They suggest, however, that a revised version might prove acceptable, if you can address their concerns. If you think that you can deal satisfactorily with the criticisms on revision, I would be pleased to see a revised manuscript. We would then return it to the reviewers.

Please ensure that you clearly highlight all changes made in the revised manuscript. Please avoid using 'Tracked changes' in Word files as these are lost in PDF conversion.

I should be grateful if you would also provide a point-by-point response detailing how you have dealt with the points raised by the reviewers in the 'Response to Reviewers' box. Please attend to all of the reviewers' comments. If you do not agree with any of their criticisms or suggestions please explain clearly why this is so.

<u>Reviewer 1</u>

*Advance summary and potential significance to field*

© 2023. Published by The Company of Biologists under the terms of the Creative Commons Attribution License (https://creativecommons.org/licenses/by/4.0/).

The manuscript by Ghosh et al describes the RILP cleavage dependent release of proinflammatory exosomes and ncRILP mediated release of antiinflammatory exosomes from donor macrophage cell lines, THP1 and RAW cells. The exosomes in turn activate transcriptional processes in target macrophages or hepatocytes, which reflect an exosome induced injury response. Thus, injured macrophages can elicit injury responses via the release of exosomes which is mediated by the cleavage of the adaptor protein RILP. The manuscript is incremental and builds on the earlier observations by the group.

*Comments for the author*

MAJOR COMMENTS:

1. The manuscript relies almost entirely on mRNA as a read out. In the one instance when protein is measured by ELISA, the increase in 3-4 fold, compared to several hundred to thousand fold reported for the mRNA. Without demonstration of an increase in the expression of protein markers the data do not support the conclusions.
2. The authors should demonstrate the activation of caspase 1 and cleavage of RILP in the donor cells both in the cell culture model and in the LPS injection model. Furthermore, it is equally important to demonstrate that the caspase 1 activation is intact in the ncRILP conditions, to place the effects downstream of inflammasome activation. Also, the crucial exosome mediated crosstalk model, should be validated using either pharmacological inhibition or genetic silencing based in vitro and in vivo studies.
3. Does the overexpression of ncRILP alter exosome number?
4. Exosome numbers and markers have not been characterized.
5. The non-carry over of LPS should be proved by testing exosome preparations for LPS.
6. Figure 3 does not demonstrate cross talk in an animal model. It would be more accurate to say that LPS activates signaling in intrahepatic leukocytes which maintain the activation response ex vivo.
7. In figure 5C, the overlapping error bars for IL-33 are very confusing given the significant reported p value.
8. Do ncRILP exosomes mitigate the effects of exosomes derived from LPS+ATP treated THP1/RAW donor cells?
9. Are there changes in hepatocyte cell death when treated with exosomes from LPS+ATP treated donor cells?
10. Authors should include cRILP overexpression as a positive control, in Fig. 2, 3, 4 too.
11. All the graphical representations should display individual datapoints.

The relative HMGB1 transcripts levels in presence of LPS+ATP seen in the range of 80,000 units (Fig. 3B) seems ambiguous. Hence, authors should validate the trend, using a total cell lysate and/or cell-supernatant WB.

12. Authors do not provide a loading control in their any of their WB panels, making it difficult to conclude. Fig. 3C presents less than 25% of exogenous cRILP-Flag expression, bu the down-stream phenotypes seem significant; hence the panel should include a WB for RILP.
13. Authors should discuss in detail on their counterintuitive inference of Fig. 5A, wherein pure exosome treatment could only match to less than half of relative IL-1ß mRNA levels induced by crude conditioned media.

MINOR COMMENTS:

1. The word "in" is missing in the second last paragraph of the introduction in the sentence: "we found that…..involved in inflammatory….
2. Arginase 1 is a urea cycle enzyme in hepatocytes. While it may be meaningful in macrophages, it cannot be a readout for anti-inflammatory effect in hepatocytes.

Reviewer 2

*Advance summary and potential significance to field*

The authors present intriguing data from a coculture model system that suggest that RILP manipulation and/or supplying ncRILP-modified exosomes from stimulated macrophages could suppress inflammatory/ injury responses (at the mRNA level) in hepatocyte target cells. The

© 2023. Published by The Company of Biologists under the terms of the Creative Commons Attribution License (https://creativecommons.org/licenses/by/4.0/).

findings suggest that RILP manipulation and/or supplying ncRILP-modified exosomes could be used as a novel therapy for the treatment of inflammatory liver diseases.
*Comments for the author*

- All blots should include a reference marker/loading control (e.g., GAPDH) and show all reactive bands.
- All qPCR experiments should include normalization to an internal reference gene. All primers used for qPCR should be included in a (supplemental) table.
- To allow comparison between experiments, the inflammatory/ injury marker panels examined should be the same in all experiments.
- Some of the inflammatory/injury markers should be validated at the protein level (cellular and/or secreted), especially in the animal model. A good one would also be the AML12 result show in figure 3B, which shows a 70,000 and 100-fold increase upon treatment with LPS and LPS/ATP, respectively. Are these large differences at the mRNA level also seen at the protein level? If not, what do these mRNA levels mean biologically/ functionally?
- Page 6 and Figure 1: The LPS/ATP activation model needs clarification. The authors state that ATP is required to activate the inflammasome (also mentioned on page 7 (5th line from below)). What is the purpose of LPS treatment? The authors should explain the roles of LPS and ATP in the assay. The results in figure 1C and D should also show the effects of LPS without ATP (that is, no inflammasome activation) in order to draw conclusions about the specific contribution of inflammasome activation. In figure 1C and D, colored boxes indicating LPS should be changed to LPS/ATP, as stated in the legend to these figure panels.
- Page 6, first paragraph: "Coculture of LPS-treated THP-1 cells with target hepatocytes (Huh7.5 or HepG2) similarly increased expression of cell injury markers such as CCL2, HMGB1, IL33, and CRP within the hepatocyte (Fig. 1C, D).".
This is not correct/precise as different markers were analyzed. Why were different inflammatory/injury markers analyzed in THP-1 cells versus hepatic cells? Why were IL33 and CCP not tested in HepG2 cells (note that IL33 was tested in the experiment depicted in figure 3D)?
- Figure 2: Also here confusion regarding LPS versus LPSA+ATP. Why are HepG2 cells treated with LPS alone (and not with ATP) and Huh7 and THP1 cells with LPS+ATP?
- Figure 2A: mCherry expression in EV-transduced cells should be shown.
Transduction efficiency (% of THP1 cells expressing mCherry or FLAG) should be shown. Loading controls should be shown.
- Page 7 and Figure 2: The statement "Like previous results, when target THP-1 cells were co-cultured with macrophages that were pretreated with LPS, the expression of IL-1β and TNFα increased dramatically in target macrophages. The expression was further increased when the target cells were co-cultured with LPS/ATP-treated producer macrophages (Fig. 2B, C). " is not correct as there are major differences. In naïve THP1 cells, the increase in TNF-alpha expression in response to stimulated empty vector-transduced THP1 cells is ~50-fold less when compared to the response to non-transduced THP1 cells (see Figure 1). Also in HepG2 cells, the increase in HMGB1 expression in response to stimulated empty vector-transduced THP1 cells was ~50-fold less when compared to the response to non-transduced THP1 cells. By contrast, IL33 expression was increased ~7-fold in empty-vector transduced cells when compared to non-transduced cells. (Figure 2C vs. 1C) It appears that the transduction of the THP1 cells with empty vector as such either dramatically inhibited or enhanced the ability of these cells to increase the expression of selective inflammatory/injury markers in target cells. In order to carefully address this, all conditions shown in figures 1 and 2 should be tested and compared with each other in the same experiment.
- Page 7 and Figure 2: It is stated that "Taken together, the data shows that the RILP cleavage status in producer cells regulates the production of signals responsible for cell-to-cell transfer of inflammatory signals. Blocking RILP cleavage dramatically inhibits pro-inflammatory cell-cell communication.".
However, RILP cleavage status in the different conditions is assumed but not shown. Moreover, the experiments did not exclude the possibility that the overexpression of RILP rather than the overexpression of a non-cleavable RILP mutant caused the inhibitory effects on inflammatory marker expression in target cells. A better control would be transduction with wild-type RILP (or another non-dominant negative, RILP mutant).
- Figure 3F. The blot appears to be cropped too tight, as lower MW bands seems to be present in at least lane 1 and 2. All bands detected on the blot with the antibody should be shown and explained.

© 2023. Published by The Company of Biologists under the terms of the Creative Commons Attribution License (https://creativecommons.org/licenses/by/4.0/).

- Figure 3E, G. Similar to the results shown in figure 2, transduction with empty vector as such appears to dramatically inhibit the gene expression response to stimulated macrophages.
Figure 4: Why were cells not treated with ATP? According to the authors (Introduction), RILP cleavage was linked to inflammasome activation, and inflammasome activation requires treatment with ATP. In the absence of inflammasome activation you would indeed not expect any differences between empty vector- and ncRILP-transduced cells. These experiments should be performed in LPS/ATP-treated cells.
Figure 4B: The label TLR4 (red box) is not correct and should be ncRILP instead.
Figure 4C-E: It is not clear relative to which value the data are presented. The y-axis in Figure 4C states "fold no treatment", but for which condition: empty vector or ncRILP? In 4C is seems both, in 4D it seems empty vector and in 4E is seems neither. This needs to be clarified.
Figure 5: There is no validation of the exosome fractions. The authors refer to an earlier paper from 2020. However, for every exosome preparation such validation should be performed.
Figure 5A: The authors should explain the difference in effect between the crude fraction and exosome fraction.
Figure 5A: The authors should explain why exosome-free medium did not evoke an inflammatory response, given that LPS-stimulated cells in all conditions secreted IL-beta (Figure 4D).
- The summary statement "Ghosh et al. describe a mechanism whereby manipulation of a Rab7 adaptor protein, RILP in macrophages prevents the packaging of inflammatory mediators into exosomes and protects hepatocytes against an injury signal.", is incorrect as the content of inflammatory mediators in exosomes of empty vector- versus ncRILP-transduced cells has not been shown.
- The title "Inflammatory macrophage to hepatocyte signals can be prevented by exosome cargo reprogramming" is not correct as no data showing exosomes cargo is shown.

<u>Reviewer 3</u>

*Advance summary and potential significance to field*

This is a straightforward manuscript describing that a non-cleavable form of a rab7 adaptor protein, RILP, prevents exosomal packaging of inflammatory cytokines thereby protecting hepatocytes from injury. The studies are well-controlled clearly presented and consistent with the authors' interpretations. Although the manuscript describes this very interesting observation, it fails to provide substantive mechanistic insight to the macrophage exosomal reprogramming described.

*Comments for the author*

RILP is known to bind dynein and rab 7. It would be useful to show/explain how the binding properties of the cleavable and noncleavable forms of RILP (cRILP and ncRILP) are altered in this process.

Also it would be useful to comment on how cRILP can both redirect lysosomes to the plasma membrane while also regulate cargo loading into exosomes. These are somewhat disparate functions -- how can one molecule can function in both of these processes? How does this relate to its binding to dynein and/or rab7 with respect to the cRILP and ncRILP forms? Do the authors have any insight into the selectivity of cargo loading into these exosomes? How does that selectivity shift?

Although the co-cultured hepatocytes and macrophages are responding to the exosomes, is it known that they are being internalized? How is the signal be received by the co-cultured cells?

Because Rab7 is known to have many functions in endocytic vesicle trafficking (e.g., endosomal maturation, late endosome to lysosome transport, and exosome release, etc.) it would be useful if the authors could comment on whether RILP functions in all or a subset of rab7 functions.

Although it is understood that the secretome includes proteins secreted via the biosynthetic pathway and exosomal release (among other things), but the word secretion is used throughout the manuscript and at times it is not always clear what form of secretion they are referring to. It might be useful to adopt more specific terms at times.

© 2023. Published by The Company of Biologists under the terms of the Creative Commons Attribution License (https://creativecommons.org/licenses/by/4.0/).

Similarly, the authors state that cRILP can redirect vesicle traffic to the plasma membrane to enhance exosome release. Do they mean lysosome redirection?
Although this might sound like splitting hairs, it was at times difficult to fully understand what the authors were referring to.

---

**First revision**

Author response to reviewers' comments

We would like to thank the reviewers for their critical and very helpful review of this manuscript. In response to the critiques, we have performed several new experiments, included data from many more controls, and rewritten various sections to include a more detailed and precise description of the experimental protocols.

The major experimental changes include a more thorough examination of inflammasome activation and RILP cleavage in producer cells. We have also included data further examining the contribution of RILP cleavage to cell-to-cell transmission of injury signals. Finally, we added a number of controls and addressed methodological issues that were either missing or needed further clarification.

A specific point-by-point response to the reviewer comments is presented below. We feel that these revisions have greatly improved the paper and appreciate the efforts of the reviewers.

**Reviewer 1**

Changes specific to reviewer 1 are noted within the manuscript as blue and green highlight.

***Major comments:***

1. The manuscript relies almost entirely on mRNA as a read out. In the one instance when protein is measured by ELISA, the increase is 3-4-fold, compared to several hundred to thousand-fold reported for the mRNA. Without demonstration of an increase in the expression of protein markers the data do not support the conclusions.
   *We performed these experiments and have now included ELISA data showing corresponding increases in several of the mRNA markers used including HMGB1 and IL33. This data is now included throughout the manuscript.*

2. The authors should demonstrate the activation of caspase 1 and cleavage of RILP in the donor cells both in the cell culture model and in the LPS injection model.
   *We agree and have now performed experiments showing caspase-1 activation in the donor cells. To confirm our findings, we also measured several downstream markers of caspase-1 activation including mature IL-1β production in donor cells as well as secretion into conditioned media. This is included in Figures 2A-B, S2, 3D-E, and 4B-C.*
   Furthermore, it is equally important to demonstrate that the caspase 1 activation is intact in the ncRILP conditions, to place the effects downstream of inflammasome activation.
   *Thank you for this important comment. To address this, we added data showing that expression of ncRILP does not alter caspase-1 activation. This is now included in Fig 5C.*
   Also, the crucial exosome mediated crosstalk model, should be validated using either pharmacological inhibition or genetic silencing based in vitro and in vivo studies.
   *We agree that incorporating pharmacological inhibition of exosome uptake would be more confirmatory. As suggested, we have now performed these experiments and show that treatment of target cells with cytochalasin D, a described extracellular vesicle inhibitor (Bastos-Amador et al., 2012; Catalano and O'Driscoll, 2020), blocks the effects of EVs. This data is presented in Fig 6E.*

3. Does the overexpression of ncRILP alter exosome number?
   *ncRILP does not change exosome number compared to control. This data was previously published in Wozniak et al., JCB 2020.*

© 2023. Published by The Company of Biologists under the terms of the Creative Commons Attribution License (https://creativecommons.org/licenses/by/4.0/). 

4. Exosome numbers and markers have not been characterized.
   *We have now added data showing Western blot analysis of extracellular vesicle preparations isolated from both control and LPS-treated THP-1 cells. Confirming previous analyses performed by our lab (Wozniak et al., 2020), Western blot analysis showed that the EV pellets contain known exosomal markers (CD63 and Flotillin) but do not contain the microvesicle marker annexin A1 or components of other organelles including the Golgi. However, to avoid any possible misrepresentation, we have elected to refer to the exosome preparations as extracellular vesicles. This data is now included in Figure S3.*

5. The non-carry over of LPS should be proved by testing exosome preparations for LPS.
   *We performed these experiments and found no carryover of LPS in the EV preparations. This data is now included in Fig 6B.*

6. Figure 4 (formerly figure 3) does not demonstrate cross talk in an animal model. It would be more accurate to say that LPS activates signaling in intrahepatic leukocytes which maintain the activation response ex vivo.
   *We agree and have amended the text accordingly. We now refer to this as an ex vivo model of animal-derived macrophages.*

7. In figure 6D (formerly Figure 5C), the overlapping error bars for IL-33 are very confusing given the significant reported p value.
   *We thank the reviewer for noting this and we apologize for inadvertently mislabeling the graph. We have corrected the figure to display the proper data.*

8. Do ncRILP exosomes mitigate the effects of exosomes derived from LPS+ATP treated THP1/RAW donor cells?
   *In this study we did not directly test whether ncRILP EVs block the effects of EVs derived from LPS or LPS/ATP treated cells.*

   NOTE: We have removed unpublished data that had been provided for the referees in confidence.

9. Are there changes in hepatocyte cell death when treated with exosomes from LPS+ATP treated donor cells?
   *We did not see cell death in hepatocytes when they were treated with EVs from LPS, or LPS/ATP treated producer THP-1 cells.*

10. Authors should include cRILP overexpression as a positive control, in Fig. 2, 3, 4 too.
    *The goal of this work is to show that ncRILP overexpression in producer cells is sufficient to modify the consequences of pathological inflammation. It is important to note that while we have showed in the past that expression of cRILP alone can mimic the effect seen when cells are treated with an inflammatory stimulus, the result does not fully recapitulate inflammasome activation. As such, using cRILP overexpression as the positive control would be misleading.*

11. All the graphical representations should display individual datapoints. The relative HMGB1 transcripts levels in presence of LPS+ATP seen in the range of 80,000 units (Fig. 3B) seems ambiguous. Hence, authors should validate the trend, using a total cell lysate and/or cell-supernatant WB.
    *We have amended the figures to show individual data points. We also included data correlating mRNA increases with protein levels. Analysis of cell lysates via ELISA are now included throughout the manuscript.*

12. Authors do not provide a loading control in their any of their WB panels, making it difficult to conclude. Fig. 3F (formally Figure 3C) presents less than 25% of exogenous cRILP-Flag

© 2023. Published by The Company of Biologists under the terms of the Creative Commons Attribution License (https://creativecommons.org/licenses/by/4.0/). 

expression, but the down-stream phenotypes seem significant; hence the panel should include a WB for RILP.

*Figure 3F shows the exogenous expression of ncRILP-FLAG in mouse-derived RAW macrophages. Western blot analysis was performed using a mouse antibody directed towards FLAG. This resulted in a non-specific band at approximately 43 kD which is present in all samples, regardless of transfection with empty vector (pCDH-EF1) or ncRILP-Flag. However, we also detected a band that only reacted with the FLAG antibody. This band is present in only the samples transfected with ncRILP-Flag. We have also added ponceau S stains and/or GAPDH to all Western blots to show equal protein loading. We have also changed the figure legend to better identify the correct band.*

13. Authors should discuss in detail on their counterintuitive inference of Fig. 6A (formerly 5A), wherein pure exosome treatment could only match to less than half of relative IL-1ß mRNA levels induced by crude conditioned media.

    *The reviewer makes an interesting observation, and several possibilities exist as to why pure EV treatment seemingly elicits a lower immune response that crude conditioned media. First, it is possible that the maximal effect (the effect seen with crude media) requires a synergy between the soluble, EV-free fraction and the EV components. Another possibility is that some of the EVs were lost during the purification. Finally, it could be an issue of dose. To control for EV numbers between treatments, we add a specific amount of EVs (5µg) to the target cell. As such, we may be dosing at a lower EV concentration than what is seen in crude media.*

***Minor comments:***

1. The word “in” is missing in the second last paragraph of the introduction in the sentence: “we found that…..involved in inflammatory….

    *We thank the reviewer for noting this omission. We have now edited the manuscript accordingly.*

2. Arginase 1 is a urea cycle enzyme in hepatocytes. While it may be meaningful in macrophages, it cannot be a readout for anti-inflammatory effect in hepatocytes.

    *Thank you for bringing this to our attention. We agree and have opted to remove this readout from the manuscript.*

**Reviewer 2**

Changes specific to reviewer 2 are noted within the manuscript as yellow and green highlight.

1. All blots should include a reference marker/loading control (e.g., GAPDH) and show all reactive bands.

    *We thank the reviewer for noting these omissions. We have now included loading controls for all western blots.*

2. All qPCR experiments should include normalization to an internal reference gene. All primers used for qPCR should be included in a (supplemental) table.

    *All qPCR data was normalized to GAPDH. We thank the reviewer for noting this oversight and we have amended the methods to include these details. We have also included a supplemental table of primers used.*

3. To allow comparison between experiments, the inflammatory/injury marker panels examined should be the same in all experiments.

    *We were not able to use the same injury markers because the different cell types (macrophages vs hepatocytes) produce different pro-inflammatory response molecules. Therefore, we used cell type specific injury markers.*

4. Some of the inflammatory/injury markers should be validated at the protein level (cellular and/or secreted), especially in the animal model. A good one would also be the AML12 result

© 2023. Published by The Company of Biologists under the terms of the Creative Commons Attribution License (https://creativecommons.org/licenses/by/4.0/).

show in figure 3B, which shows a 70,000 and 100-fold increase upon treatment with LPS and LPS/ATP, respectively. Are these large differences at the mRNA level also seen at the protein level? If not, what do these mRNA levels mean biologically/ functionally?

*We performed ELISAs on the target cell lysates and show corresponding increases in the protein levels which correlate to several of the mRNA markers used including HMGB1 and IL33. We do note that the increases in intracellular cytokine protein levels are not of the same magnitude as those seen via PCR. This is likely due to the fact that cytokines are actively and rapidly secreted and by measuring intracellular cytokine levels, we are catching only that which has not yet been secreted. Nonetheless, the ELISA data does confirm our qPCR findings that target cells respond to inflammatory macrophages by increasing cytokines involved in injury responses. This data is now included throughout the manuscript.*

5. Page 6 and Figure 1: The LPS/ATP activation model needs clarification. The authors state that ATP is required to activate the inflammasome (also mentioned on page 7 (5th line from below)). What is the purpose of LPS treatment? The authors should explain the roles of LPS and ATP in the assay. The results in figure 1C and E (formerly Figs 1C and D) should also show the effects of LPS without ATP (that is, no inflammasome activation) in order to draw conclusions about the specific contribution of inflammasome activation. In figure 1C and D, colored boxes indicating LPS should be changed to LPS/ATP, as stated in the legend to these figure panels.

   *In previous studies performed in the lab used a short-term "2-hit" method of inflammasome activation whereby cells were treated with a higher dose of LPS (1ug/ml) for 3 hours followed by ATP for another hour. During this short course of treatment, each component alone is not sufficient to activate the inflammasome and both LPS and ATP are required to fully activate the inflammasome. This study initially set out to use the LPS/ATP combo as the standard method but due to toxicity in some experiments, particularly THP-1-HepG2 co-culture, we chose to use a long-term treatment with a lower dose of LPS (100ng/ml). This longer period is sufficient to fully activate the inflammasome without the addition of ATP (Dalby, 2018; Monguió-Tortajada et al., 2018). We fully agree with the reviewer that the addition of LPS/ATP co-treatment adds confusion to the findings and have thus decided to remove this from the manuscript.*

   *To confirm that a long-term treatment with a lower dose of LPS (100ng/ml) can induce inflammasome activation without the addition of ATP, we measured both the intracellular level of cleaved caspase-1 and the amount of IL-1β secreted from LPS treated producer cells. We chose these as they are direct markers of inflammasome activation. We found significant increases in both intracellular cleaved caspase-1 as well as secreted IL-1β when THP-1 cells were treated with 100ng/ml LPS for 24 hours. This data is now included in Figure S1, 4A, and 5. We have also included data showing that this treatment results in RILP cleavage. This is included in Figures 2A-B, S2, 3D-E, and 4B-C.*

6. Page 6, first paragraph: "Coculture of LPS-treated THP-1 cells with target hepatocytes (Huh7.5 or HepG2) similarly increased expression of cell injury markers such as CCL2, HMGB1, IL33, and CRP within the hepatocyte (Fig. 1C, E) (formerly Fig 1C, D).". This is not correct/precise as different markers were analyzed. Why were different inflammatory/injury markers analyzed in THP-1 cells versus hepatic cells? Why were IL33 and CCP not tested in HepG2 cells (note that IL33 was tested in the experiment depicted in figure 3D)?

   *As stated previously in comment 3, we were unable to use the same injury markers for each cell type because the different cells (macrophages vs hepatocytes) produce different pro-inflammatory response molecules. Therefore, we used cell-specific injury markers. We do not have CRP data for HepG2 because detectable levels were not seen by PCR. To clarify this point, we have now added details regarding the use of cell-type specific injury markers within the text.*

7. Figure 2: Also, here confusion regarding LPS versus LPSA+ATP. Why are HepG2 cells treated with LPS alone (and not with ATP) and Huh7 and THP1 cells with LPS+ATP?

   *We agree that we did not fully explain why we did not treat HepG2 cells with ATP. Please see comment 5 above for more details.*

© 2023. Published by The Company of Biologists under the terms of the Creative Commons Attribution License (https://creativecommons.org/licenses/by/4.0/). 

8. Figure 2c (formerly 2A): mCherry expression in EV-transduced cells should be shown. Transduction efficiency (% of THP1 cells expressing mCherry or FLAG) should be shown. Loading controls should be shown.

   *The mCherry-based lentiviral plasmids have been extensively used and characterized in previous work from our lab (Wozniak et al., PLoS Pathog. 2010). Expression is consistent between the empty vector and mCherry plasmids carrying genes of interest. Also, as the reviewer noted in comment 9, when lentivirus is used to transduce producer cells, the response in the target cells is not of the same magnitude as when no lentivirus is used. Because this magnitude change occurs with both mCherry empty vector and mCherry-ncRILP-Flag, we feel this corroborates equal expression of the two plasmids.*

9. Page 7 and Figure 2: The statement "Like previous results, when target THP-1 cells were co-cultured with macrophages that were pretreated with LPS, the expression of IL-1ß and TNFα increased dramatically in target macrophages. The expression was further increased when the target cells were co-cultured with LPS/ATP-treated producer macrophages (Fig. 2D, E) (formerly 2B, C). " is not correct as there are major differences. In naïve THP1 cells, the increase in TNF-alpha expression in response to stimulated empty vector-transduced THP1 cells is ~50-fold less when compared to the response to non-transduced THP1 cells (see Figure 1). Also, in HepG2 cells, the increase in HMGB1 expression in response to stimulated empty vector-transduced THP1 cells was ~50-fold less when compared to the response to non-transduced THP1 cells. By contrast, IL33 expression was increased ~7-fold in empty-vector transduced cells when compared to non-transduced cells. (Figure 2C vs. 1C) It appears that the transduction of the THP1 cells with empty vector as such either dramatically inhibited or enhanced the ability of these cells to increase the expression of selective inflammatory/injury markers in target cells. In order to carefully address this, all conditions shown in figures 1 and 2 should be tested and compared with each other in the same experiment.

   *When lentivirus is used to transduce producer cells, the response in the target cell is not of the same magnitude as when no lentivirus is used. However, it is important to note that significant increases in target cell injury markers are still seen when producer cells are treated with LPS. Even more, these increases are prevented when the producer cell is transduced with ncRILP-expressing lentivirus. Therefore, the decrease in magnitude seen does not negate the effect of ncRILP expression and overall conclusions of this paper.*

10. Page 7 and Figure 2: It is stated that "Taken together, the data shows that the RILP cleavage status in producer cells regulates the production of signals responsible for cell-to-cell transfer of inflammatory signals. Blocking RILP cleavage dramatically inhibits pro-inflammatory cell-cell communication." However, RILP cleavage status in the different conditions is assumed but not shown. Moreover, the experiments did not exclude the possibility that the overexpression of RILP rather than the overexpression of a non-cleavable RILP mutant caused the inhibitory effects on inflammatory marker expression in target cells. A better control would be transduction with wild-type RILP (or another, non-dominant negative, RILP mutant).

    *We have now performed experiments showing that in producer cells, RILP is cleaved after treatment with LPS. This data is shown in Figures 2A-B, S2, 3D-E, and 4B-C. We have also edited the text to reflect the reviewer's suggestion regarding RILP cleavage status.*

11. In Figure 4E (formally Figure 3F). The blot appears to be cropped too tight, as lower MW bands seems to be present in at least lane 1 and 2. All bands detected on the blot with the antibody should be shown and explained.

    *Figure 4E shows the exogenous expression of ncRILP-FLAG in CD11b-derived mouse macrophages. Western blot analysis was performed using a mouse antibody directed towards FLAG. This resulted in a non-specific band at approximately 43 kD which is present in all samples, regardless of transfection with empty vector (pCDH-EF1) or ncRILP-Flag. However, we also detected a band that only reacted with the FLAG antibody. This band is present in only the samples transfected with ncRILP-Flag. We have also added ponceau S stain to show equal protein loading. We have also changed the figure legend to better identify the correct band.*

© 2023. Published by The Company of Biologists under the terms of the Creative Commons Attribution License (https://creativecommons.org/licenses/by/4.0/).

12. Figure 3D, F (formerly 3E, G). Similar to the results shown in figure 2, transduction with empty vector as such appears to dramatically inhibit the gene expression response to stimulated macrophages.
    *This comment was previously addressed in comment 9.*

13. Figure 5 (formerly Fig 4): Why were cells not treated with ATP? According to the authors (Introduction), RILP cleavage was linked to inflammasome activation, and inflammasome activation requires treatment with ATP. In the absence of inflammasome activation you would indeed not expect any differences between empty vector- and ncRILP-transduced cells. These experiments should be performed in LPS/ATP-treated cells.
    *As stated in a prior comment (see comment 5), we chose to use a long-term treatment with a lower dose of LPS (100ng/ml). This longer period is sufficient to fully activate the inflammasome without the addition of ATP (Dalby, 2018; Monguió-Tortajada et al., 2018). To eliminate confusion, we have omitted all experiments using LPS/ATP.*

14. Figure 5B (formerly 4B): The label TLR4 (red box) is not correct and should be ncRILP instead.
    *We thank the reviewer for noting this oversight and have corrected this.*

15. Figure 5D-F (formerly 4C-E): It is not clear relative to which value the data are presented. The y-axis in Figure 4C states "fold no treatment", but for which condition: empty vector or ncRILP? In 4C is seems both, in 4D it seems empty vector and in 4E is seems neither. This needs to be clarified.
    *For each experiment, the data are presented as fold change in respect to their own untreated. We have amended this in the figure axes.*

16. Figure 6 (formerly figure 5): There is no validation of the exosome fractions. The authors refer to an earlier paper from 2020. However, for every exosome preparation such validation should be performed.
    *We have now added data showing Western blot analysis of extracellular preparations isolated from both control and LPS-treated THP-1 cells. Confirming previous analyses performed by our lab (Wozniak et al., 2020), Western blot analysis showed that the EV pellets contain known exosomal markers (CD63 and Flotillin) but do not contain the microvesicle marker annexin A1 or components of other organelles including the Golgi. However, to avoid any possible misrepresentation, we have elected to refer to the exosome preparations as extracellular vesicles. This data is now included in Figure S3.*

17. Figure 6A (formerly 5A): The authors should explain the difference in effect between the crude fraction and exosome fraction.
    *Cell culture supernatants were subjected to differential centrifugation as stated in Thery et al., 2006. The crude fraction represents the sample after the 10,000xg spin. This contains both soluble cytokines and EVs. After centrifugation at 100,000xg, the supernatant representing the EV-free soluble fraction was removed. The EV pellet was washed in PBS and again spun at 100,000xg. This final pellet represents EV fraction. We have now added a schematic in Figure S3.*

18. Figure 6A (formerly 5A): The authors should explain why exosome-free medium did not evoke an inflammatory response, given that LPS-stimulated cells in all conditions secreted IL-beta (Figure 4F).
    *The reviewer makes an astute observation central to the conclusion of this paper. The EV-free soluble fraction alone is not sufficient to elicit an immune response in target cells, yet treatment with EVs is. This finding suggests that IL-1β (or other soluble factors) are not responsible for eliciting immune responses in this inflammatory model. However, EVs are.*

19. The summary statement "Ghosh et al. describe a mechanism whereby manipulation of a Rab7 adaptor protein, RILP in macrophages prevents the packaging of inflammatory mediators into exosomes and protects hepatocytes against an injury signal.", is incorrect as the content of inflammatory mediators in exosomes of empty vector- versus ncRILP-transduced cells has not been shown.

© 2023. Published by The Company of Biologists under the terms of the Creative Commons Attribution License (https://creativecommons.org/licenses/by/4.0/).

*We agree and have amended the summary statement.*

20. The title "Inflammatory macrophage to hepatocyte signals can be prevented by exosome cargo reprogramming" is not correct as no data showing exosomes cargo is shown.
*We noted the reviewer's suggestion and have modified the title.*

**Reviewer 3**

Changes specific to reviewer 2 are noted within the manuscript as pink highlight.

1. RILP is known to bind dynein and rab 7. It would be useful to show/explain how the binding properties of the cleavable and non-cleavable forms of RILP (cRILP and ncRILP) are altered in this process.
*RILP (Rab interacting lysosomal protein) is a key regulator of endo-lysosomal trafficking. It interacts with Rab7 through its C-terminal domain while its N-terminal domain recruits the dynein-dynactin motor complex. RILP is therefore responsible for directing minus-end directed microtubule transport of Rab7-containing vesicles from the endosome to the multivesicular body (MVB) and finally the lysosome. Our lab discovered that RILP is modified by a caspase-1 mediated proteolytic cleavage event that liberates it from dynein. This cleavage generates a C-terminal fragment of the protein. Cleaved RILP (cRILP) reroutes endocytic vesicular trafficking away from the lysosome and instead the vesicle moves towards the plasma membrane. This leads to an enhancement of plasma membrane fusion events and accounts for a burst of vesicular secretion including the secretion of EVs. We have now added text within the introduction explaining the Rab7/RILP axis and how RILP cleavage alters the trafficking of Rab7-containing vesicles.*

1. It would be useful to comment on how cRILP can both redirect lysosomes to the plasma membrane while also regulate cargo loading into exosomes. These are somewhat disparate functions -- how can one molecule can function in both of these processes? How does this relate to its binding to dynein and/or rab7 with respect to the cRILP and ncRILP forms? Do the authors have any insight into the selectivity of cargo loading into these exosomes? How does that selectivity shift?
*The reviewer asks an important question and one currently under investigation in the lab. The cleavage of RILP results in a truncated protein that binds Rab7 but cannot make the link to the dynein complex. As a result, Rab7-containing vesicles such as the late endosome/MVB do not reach the lysosome and are instead redirected to the plasma membrane. In short, it is not the lysosome that is redirected to the plasma membrane but the Rab7-containing vesicles that exist preceding the lysosomes (the late endosome/MVB) that are affected. On the opposite hand, ncRILP binds both Rab7 and dynein and thus trafficking to the lysosome still occurs. However, it occurs more efficiently and rapidly. We demonstrated this effect using HCV infection as a model of inflammation (Wozniak et al., PNAS 2016).*

*Regarding how the cleavage status of RILP can regulate the selectivity of cargo loading into exosomes: In our publication (Wozniak et al., 2020) we describe the differential recruitment of proteins involved in exosome biogenesis and loading during inflammation. We reported that while both cRILP and ncRILP interact with the RNA binding protein FMR1, only cRILP will recruit Hrs, a component of the ESCRT-0 complex. This complex associates with the MVB and is responsible for recognizing and loading cargo into the exosome. We further detail how the cRILP-FMR1-Hrs complex uniquely loads only "pro-inflammatory" miRNAs and show that FMR1 binds to miRNAs containing a specific AAUGC motif. When ncRILP is expressed, we see differential miRNA loading, specifically a de-enrichment of pro-inflammatory miRNAs within the exosome.*

2. Although the co-cultured hepatocytes and macrophages are responding to the exosomes, is it known that they are being internalized? How is the signal be received by the co-cultured cells?

© 2023. Published by The Company of Biologists under the terms of the Creative Commons Attribution License (https://creativecommons.org/licenses/by/4.0/). 

*The author makes an important observation in that we did not originally confirm that the target cells are taking up the exosomes. We have now added data incorporating the pharmacological inhibition of exosome uptake. The data show that treatment of target cells with cytochalasin D, a described extracellular vesicle inhibitor (Bastos-Amador et al., 2012; Catalano and O'Driscoll, 2020), blocks the effects of EVs on target cells. This data is presented in Fig 6E.*

*At this point we do not know how the EV signal is being received by the target cells or what promotes EV entry.*

3. Because Rab7 is known to have many functions in endocytic vesicle trafficking (e.g., endosomal maturation, late endosome to lysosome transport, and exosome release, etc.) it would be useful if the authors could comment on whether RILP functions in all or a subset of rab7 functions.
   *We touched on this in comment 2 from this reviewer. RILP cleavage affects any trafficking process that requires Rab7, specifically the trafficking of the late endosome/MVB to the lysosome. In previous work from our lab, we show that after cleavage cRILP no longer binds dynein and the MVB is redirected to plasma membrane where its contents are secreted. Because the MVB is the site of exosome biogenesis, cRILP also affects exosome release. We have amended the manuscript to clarify the functions of both RILP and Rab7 in vesicular trafficking.*

4. Although it is understood that the secretome includes proteins secreted via the biosynthetic pathway and exosomal release (among other things), but the word secretion is used throughout the manuscript and at times it is not always clear what form of secretion they are referring to. It might be useful to adopt more specific terms at times.
   *We thank the reviewer for noting this confusion. We have amended the manuscript to specifically delineate secretory phenomena whether it be EV-mediated or cytokine/biosynthetic-mediated.*

5. Similarly, the authors state that cRILP can redirect vesicle traffic to the plasma membrane to enhance exosome release. Do they mean lysosome redirection? Although this might sound like splitting hairs, it was at times difficult to fully understand what the authors were referring to.
   *We thank the reviewer for bringing this to our attention. When we refer the redirection of vesicles, we mean the late endosome/MVB, not the lysosome. We have amended the manuscript accordingly.*

---

Second decision letter

MS ID#: JOCES/2022/260691

MS TITLE: Inflammatory macrophage to hepatocyte signals can be prevented by extracellular vesicle reprogramming

AUTHORS: Priyanka Ghosh, Kyo Sasaki, Isabel Aranzazu Pulido Ruiz, Kayla E King, Steven A Weinman, and Ann L Wozniak
ARTICLE TYPE: Research Article

I am happy to tell you that your manuscript has been accepted for publication in Journal of Cell Science, pending standard ethics checks.

Reviewer 1

*Advance summary and potential significance to field*

© 2023. Published by The Company of Biologists under the terms of the Creative Commons Attribution License (https://creativecommons.org/licenses/by/4.0/). 

This manuscript describes the inflammasome-mediated activation of the protein RLIM within macrophages which leads to the release of proinflammatory extracellular vesicles. These vesicles elicit inflammatory responses in hepatocytes. While the release of proinflammatory extracellular vesicles from macrophages has been described, the mechanistic advances in this manuscript are novel and of value to the field. Overreliance on LPS limits generalizability, and is likely relevant to severe alcoholic hepatitis and not the other liver diseases.

*Comments for the author*

My comments have been addressed satisfactorily.

<u>Reviewer 2</u>

*Advance summary and potential significance to field*

see first review.

*Comments for the author*

The authors have addressed my earlier comments satisfactorily.

<u>Reviewer 3</u>

*Advance summary and potential significance to field*

This is a straightforward manuscript describing that a non-cleavable form of a rab7 adaptor protein, RILP, prevents exosomal packaging of inflammatory cytokines thereby protecting hepatocytes from injury. The studies are well-controlled clearly presented and consistent with the authors' interpretations.

*Comments for the author*

The authors have adequately addressed my concerns.

© 2023. Published by The Company of Biologists under the terms of the Creative Commons Attribution License (https://creativecommons.org/licenses/by/4.0/).
